# Asymmetric Synthesis of US-FDA Approved Drugs over Five Years (2016–2020): A Recapitulation of Chirality

**DOI:** 10.3390/ph16030339

**Published:** 2023-02-22

**Authors:** Rekha Tamatam, Dongyun Shin

**Affiliations:** 1 College of Pharmacy, Gachon University, 191 Hambakmoe-ro, Yeonsu-gu, Incheon 21936, Republic of Korea; 2 Gachon Pharmaceutical Research Institute, Gachon University, 191 Hambakmoe-ro, Yeonsu-gu, Incheon 21936, Republic of Korea

**Keywords:** FDA, approve, drug, chiral, asymmetric, synthesis

## Abstract

Chirality is a major theme in the design, discovery, and development of new drugs. Historically, pharmaceuticals have been synthesized as racemic mixtures. However, the enantiomeric forms of drug molecules have distinct biological properties. One enantiomer may be responsible for the desired therapeutic effect (eutomer), whereas the other may be inactive, interfere with the therapeutic form, or exhibit toxicity (distomer). Classical chemical synthesis usually leads to a racemic mixture unless stereospecific synthesis is employed. To meet the requirements of single-enantiomeric drugs, asymmetric synthesis has evolved at the forefront of drug discovery. Asymmetric synthesis involves the conversion of an achiral starting material into a chiral product. This review emphasizes the methods used for synthesizing FDA-approved chiral drugs during 2016–2020, with a special focus on asymmetric synthesis by means of chiral induction, resolution, or chiral pool.

## 1. Introduction

### 1.1. Background

**Figure 1:**
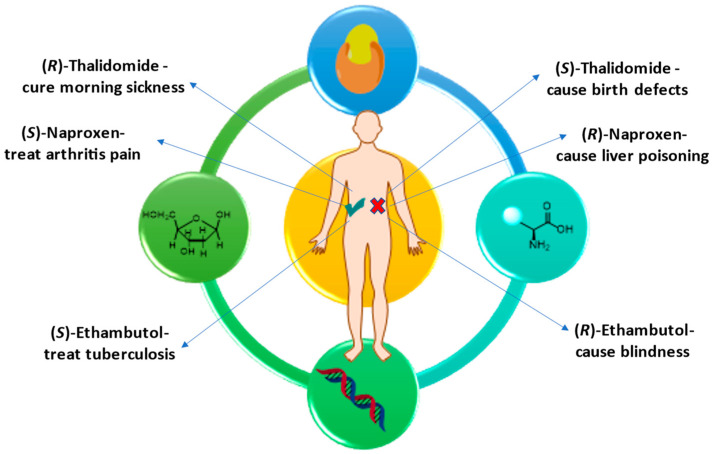
Existence of chirality in the human body.

Chirality is an all-encompassing phenomenon [1]. The earliest scientific evidence for chirality was found by Biot in 1815 while discovering the optical rotation of camphor [2]. In 1848, Louis Pasteur discovered that two tartaric acid molecules with the same properties differed in the sign of their optical rotation [3]. In general, this fundamental discovery was the basis for the development of stereochemistry and, particularly, the phenomenon of “chirality”. In nature, both macroscopic as well as microscopic objects can be chiral [4]. A molecule is described as chiral when it can exist in two forms, enantiomers, which have the same chemical structure but are non-superimposable mirror images of each other [5]. The human body is naturally composed of chiral amino acids, sugars, enzymes or receptors, and nucleic acids. Enzymes that are chiral only bind to the enantiomer that has the exact groups that fit into their binding site. Hence, each enantiomer, with respect to its configuration, has a specific action in the body and is selectively metabolized [6]. For instance, thalidomide was initially sold as a racemic drug for the treatment of women with morning sickness; however, it also had a teratogen effect and was subsequently withdrawn from the market. The *R*-enantiomer showed a positive therapeutic effect, whereas the *S*-enantiomer resulted in the development of birth defects [7] (Figure 1).

### 1.2. Chirality in FDA Drugs

Our understanding of the concept of chirality has played an essential role in the application of chiral bioactive compounds in pharmaceuticals, agrochemicals, flavors, and fragrances [8,9,10]. Chiral drugs offer several benefits, including that (1) chiral drugs can have higher potency compared to their non-chiral counterparts due to better pharmacokinetic and pharmacodynamic properties; (2) by isolating a specific enantiomer, chiral drugs can reduce unwanted side effects; (3) chiral drugs can enhance drug delivery by targeting specific enzymes or receptors; and (4) using a single enantiomer can reduce the cost of production and lead to lower drug prices for patients. In recent years, the trend in chiral drug discovery has shifted toward the development of single enantiomer drugs. This trend is driven by several factors: increased understanding of the importance of chirality in drug action and toxicity; advancements in analytical techniques that enable the separation and characterization of enantiomers; the need for more effective and safer drugs, as well as the potential for improved patent protection and market exclusivity; and increased regulatory focus on the safety and efficacy of chiral drugs. Overall, the trend in chiral drug discovery is toward the development of single enantiomer drugs, which offer the potential for improved safety, efficacy, and cost-effectiveness.

Since the FDA’s 1992 policy [11], the synthesis of single enantiomers has gained more attention than that of racemic drugs. About 56% of the pharmaceuticals available in the market and used in therapy are chiral, and amongst those drugs, 88% are administered as racemates [12]. As of 2001, racemic drugs can no longer be registered. As drugs can have structural homology across similar biological targets [13], it is widely believed that knowledge of new chemical entities and approaches to their construction will enhance our ability to discover new drugs more efficiently. In order to reduce the toxicity and side effects associated with the inactive enantiomer, the synthesis of enantiomerically pure compounds is essential. In this context, chirality has become an important challenge in the synthesis of drugs [14]. This review presents the synthetic routes for 89 new molecular entities approved by the FDA within the period of 2016–2020. The synthetic sequences described in this review have all been previously reported in patents or articles and represent the process scale or discovery routes of potential chiral drugs. In recent years, the number of chiral drugs approved has been growing in number. For instance, 20 out of the 35 pharmaceuticals approved by the FDA in 2020 are chiral [15]. A chiral drug can be synthesized from commercially available substrates (with stereocenters) or a chiral pool (naturally occurring substrates). Other ways to develop a chiral drug candidate are by employing a chiral auxiliary, using a chiral reagent, or by resolving the racemic precursor. The purpose of this review article is to analyze the recent chiral drugs and highlight the importance of asymmetric synthesis in biologically active compounds, and pharmaceuticals in particular.

## 2. Methodology

**Figure 2:**
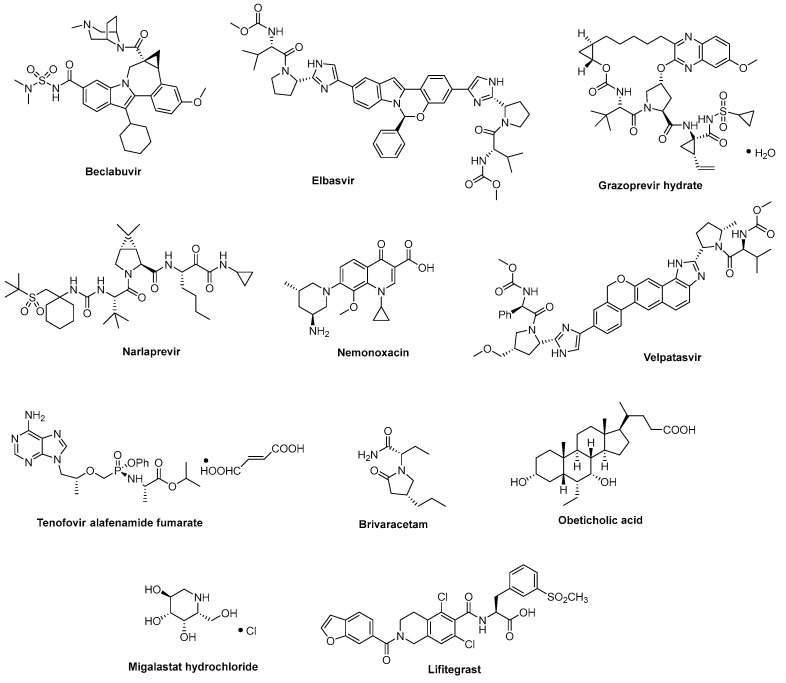
Structure of chiral drugs approved in 2016.

**Figure 3:**
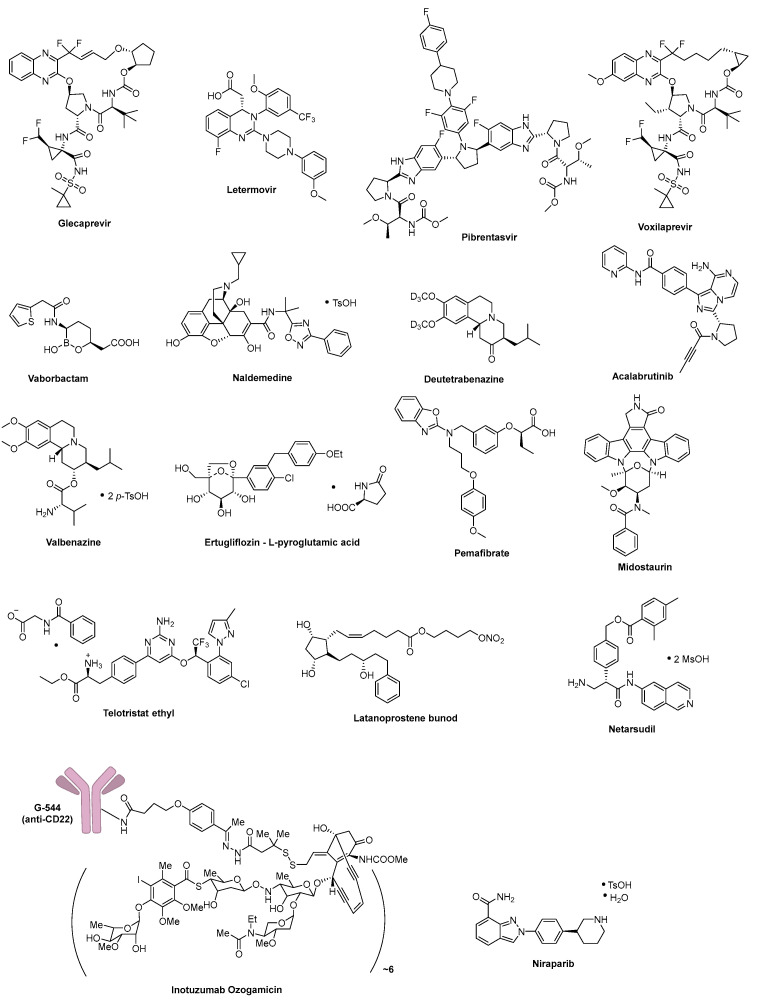
Structure of chiral drugs approved in 2017.

**Figure 4:**
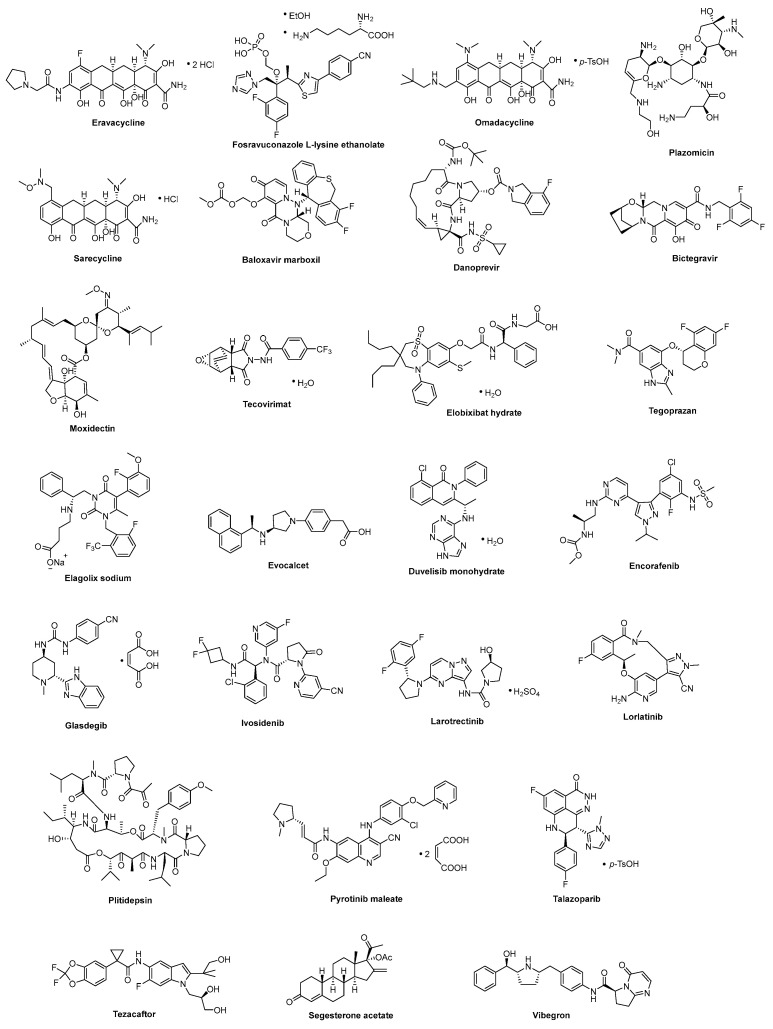
Structure of chiral drugs approved in 2018.

**Figure 5:**
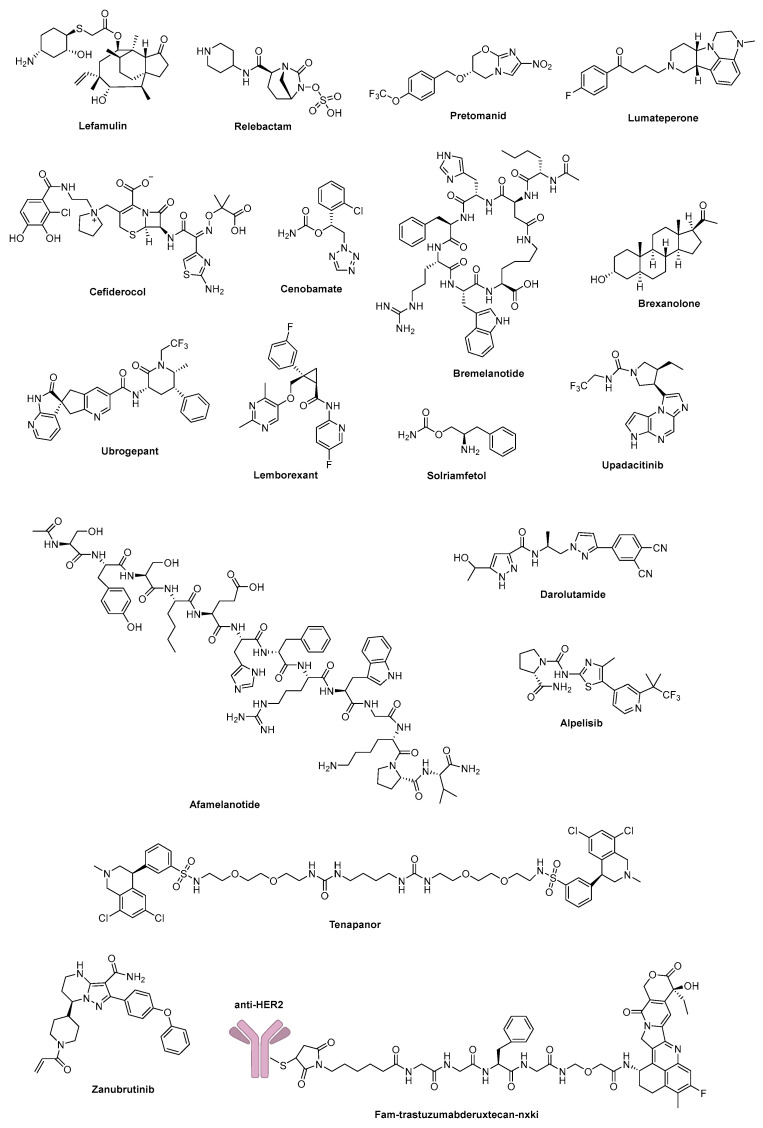
Structure of chiral drugs approved in 2019.

**Figure 6:**
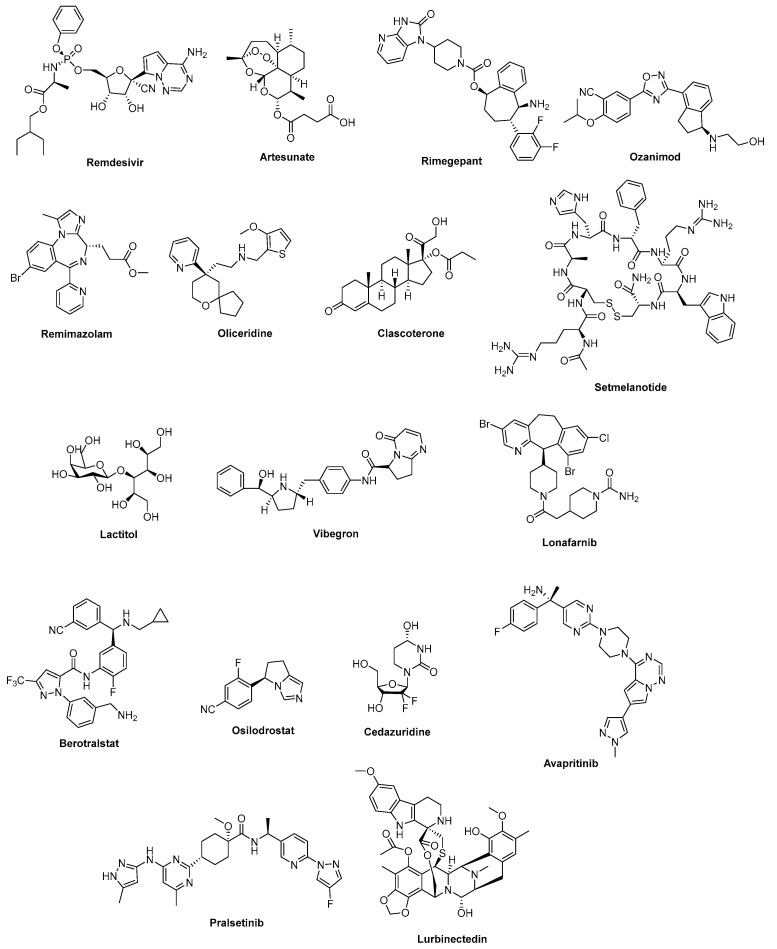
Structure of chiral drugs approved in 2020.

Data for the study were collected from the online database of the FDA under the category of novel drug approval from 2016 to 2020. All drugs are listed under the following parameters: name of the drug, active ingredient, pharmaceutical class of drug, indication for use in the patient, and number of chiral centers. In addition, a literature search was conducted using electronic databases such as PubMed, Annual Reports, Science Direct, and Drug Bank. The structures of the chiral drugs by year are shown in Figure 2, Figure 3, Figure 4, Figure 5 and Figure 6.

**Figure 7:**
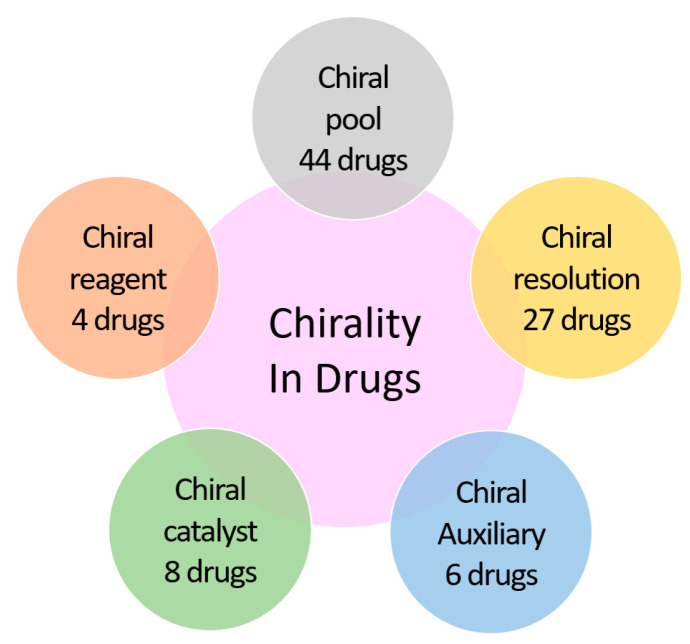
Classification of drugs on basis of their chiral induction.

These drugs were categorized based on the method by which chirality was induced during the manufacturing process, as follows (Figure 7):

1. Chiral pool approach: Synthesis from naturally occurring or chiral substrates;
2. Chiral resolution: Resolving the racemic mixture at any stage of synthesis;
3. Asymmetric synthesis: By adopting a chiral auxiliary, a chiral catalyst, or a chiral reagent.

Chirality can be induced at any stage, either as a fragment or from an achiral substrate. Due to the large size of the dataset, only the step where chirality is being introduced is discussed here. Starting from drugs with one chiral center, a year-wise classification is presented.

## 3. Discussion

### 3.1. Drugs with One Chiral Center

#### 3.1.1. Lifitegrast (2016)

**Scheme 1:**
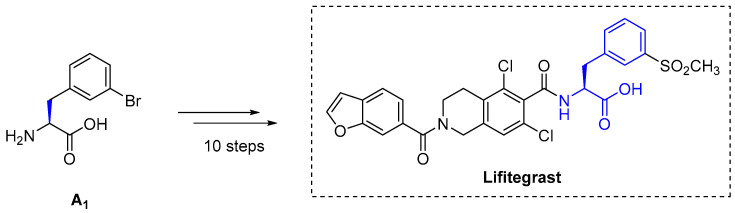
Synthesis of Lifitegrast.

Lifitegrast, developed by SARcode Bioscience (Brisbane, CA 94005 USA) [16], is marketed for the treatment of dry eye disease [17]. The total synthesis of Lifitegrast is carried out in 10 steps, starting from the commercially available chiral substrate 3-bromo-l-phenylalanine (**A_1_**). Lifitegrast is obtained in 88% yield (Scheme 1) [18].

#### 3.1.2. Acalabrutinib (2017)

**Scheme 2:**
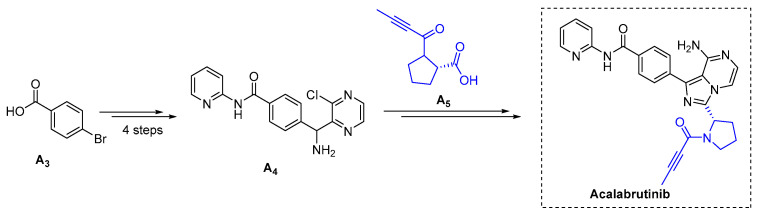
Synthesis of Acalabrutinib.

Acalabrutinib, developed by Acerta Pharma, is used for the treatment of mantle cell lymphoma (MCL) [19]. Starting from the 4-bromo benzoic acid (**A_3_**), four steps are carried out to obtain the intermediate, **A_4_**. Commercially available chiral proline derivative, **A_5_**, undergoes amidation with **A_4_** via acid chloride to yield acalabrutinib in 86% yield (Scheme 2) [20,21].

#### 3.1.3. Pemafibrate (2017)

**Scheme 3:**
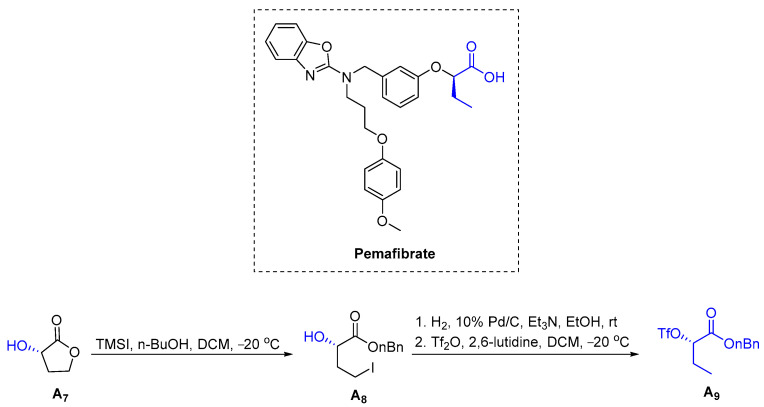
Synthesis of Pemafibrate.

Pemafibrate, developed by Kowa Pharmaceuticals, is used for the treatment of hyperlipidemia [22]. Chiral fragment **A_9_** is synthesized from enantiopure (*S*)-2-hydroxybutyrolactone (**A_7_**) via a ring-opening reaction in the presence of trimethylsilyl iodide. The hydrogenative reduction of A_8_ and triflate addition using 2,6-lutidine produces **A_9_**. **A_9_** undergoes inversion of the configuration using potassium carbonate and acetonitrile, resulting in the formation of pemafibrate in 75% yield (Scheme 3) [23].

#### 3.1.4. Letermovir (2017)

**Scheme 4:**
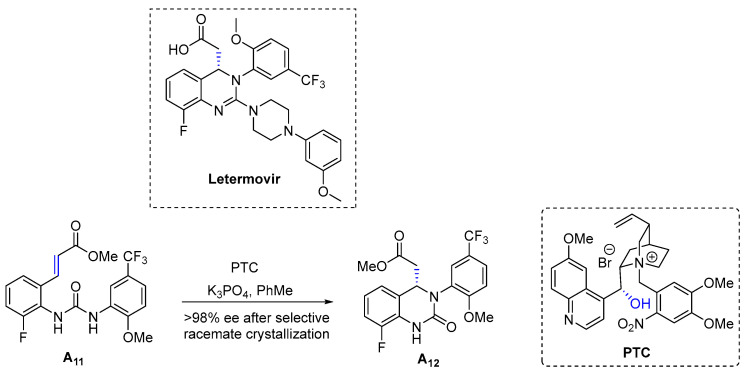
Synthesis of Letermovir.

AiCuris developed letermovir for the treatment of cytomegalovirus infections [24]. A stereogenic center is installed in letermovir during the urea cyclization step. Urea (**A_11_**) undergoes cyclization in a biphasic mixture of aqueous K_3_PO_4_ and toluene in the presence of a cinchona-alkaloid-based phase-transfer catalyst and results in the formation of quinazolinone racemate (**A_12_**) (Scheme 4) [25].

#### 3.1.5. Netarsudil (2017)

**Scheme 5:**
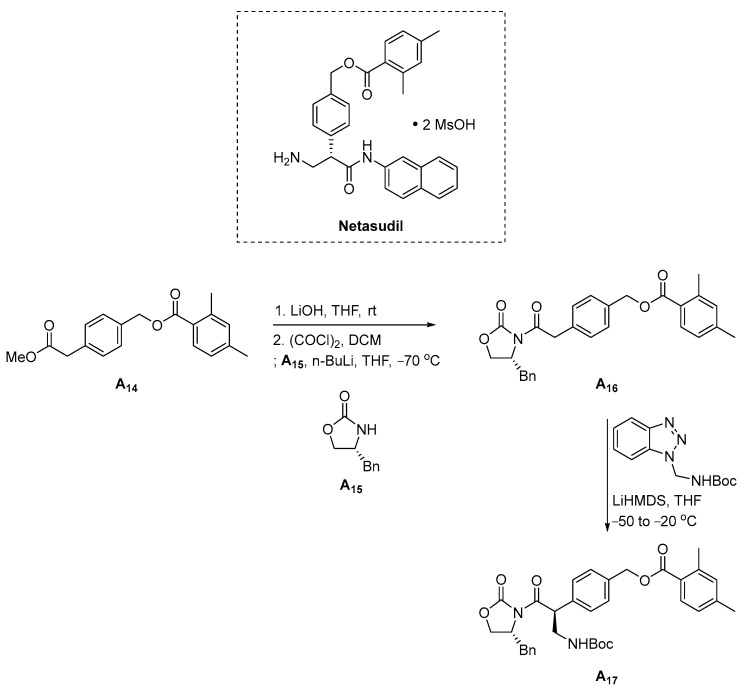
Synthesis of Netarsudil.

Aerie Pharmaceuticals developed netarsudil for the treatment of ocular hypertension [26]. The Evans oxazolidinone (**A_15_**) acts as a chiral auxiliary and is later removed in the presence of hydrogen peroxide. Diester **A_14_** undergoes regioselective hydrolysis, followed by acid chloride formation and amidation to give the ester derivative, **A_16_**. Treatment of Evans oxazolidinone compounds with LiHMDS and Boc-protected benzotriazolylmethylamine produces **A_17_**, through which essential stereochemistry for netarsudil is installed (Scheme 5) [27].

#### 3.1.6. Niraparib (2017)

**Scheme 6:**
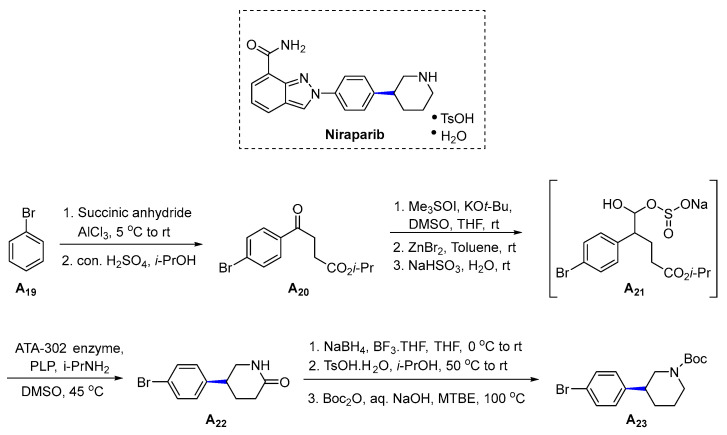
Synthesis of Niraparib.

Niraparib, discovered by Merck & Co. (Rahway, NJ, USA) and developed by Tesaro, is used for the treatment of peritoneal cancer [28]. Niraparib, having one chiral center, is obtained from piperidine subunit **A_23_**, which is synthesized from bromobenzene (**A_19_**). The bisulfite adduct, A_21_, undergoes a transaminase reaction catalyzed by the ATA-302 enzyme and co-catalyzed by pyridoxal-5-phosphate (PLP), yielding chiral piperidine (**A_22_**) (Scheme 6) [29,30,31,32].

#### 3.1.7. Lorlatinib (2018)

**Scheme 7:**
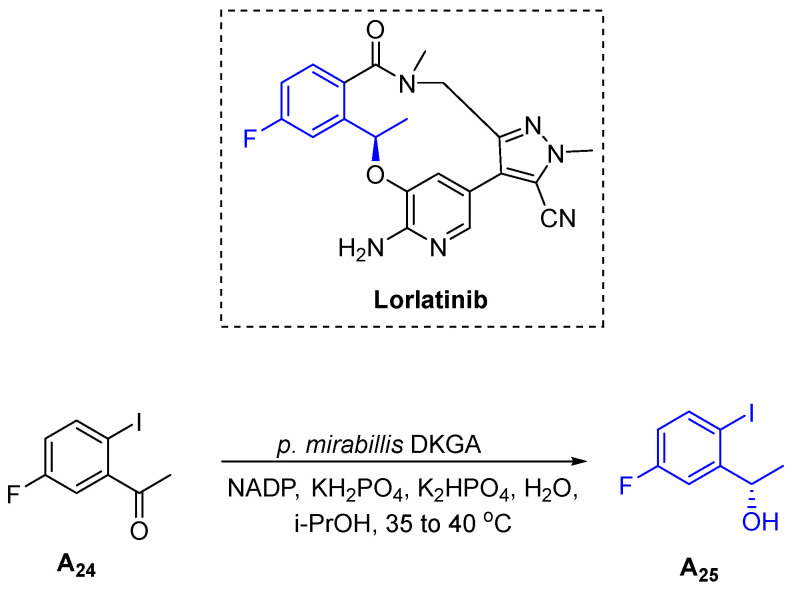
Synthesis of Lorlatinib.

Lorlatinib, developed by Pfizer, is an oncological drug [33]. Lorlatinib, a macrocycle, is constructed from chiral alcohol intermediate **A_25_**. This key step involves enantioselective reduction using a biocatalyst (Scheme 7) [34].

#### 3.1.8. Elobixibat Hydrate (2018)

**Scheme 8:**
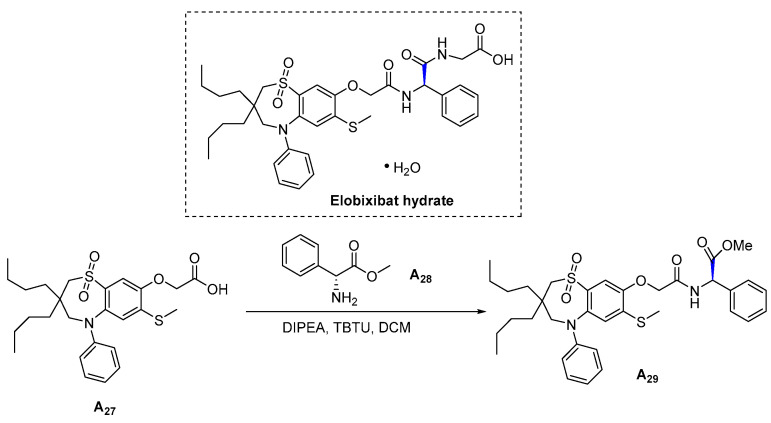
Synthesis of Elobixibat hydrate.

EA Pharma and Mochida developed elobixibat hydrate for the treatment of chronic idiopathic constipation [35]. The single chiral carbon is generated during acid-ester coupling in the presence of TBTU and methyl (R)-2-amino-2-phenylacetate (**A_28_**) (Scheme 8) [36].

#### 3.1.9. Tezacaftor (2018)

**Scheme 9:**
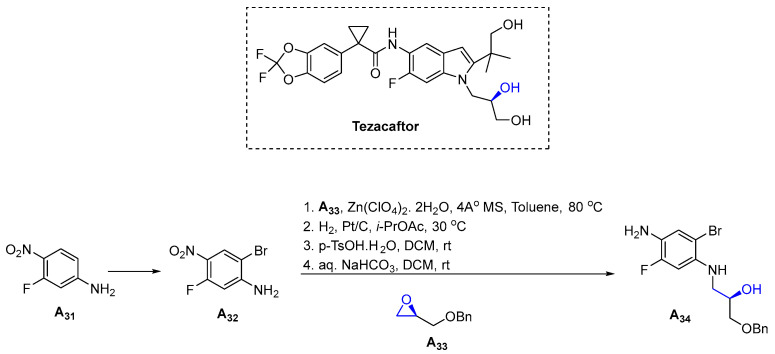
Synthesis of Tezacaftor.

Discovered and developed by Vertex Pharmaceuticals [37,38], tezacaftor is a broad-acting cystic fibrosis transmembrane conductance regulator (CFTR). The scaled synthesis of tezacaftor (Scheme 9) [39] begins with 3-fluoro-4-nitroaniline (**A_31_**). Regioselective bromination with N-Bromosuccinimide (NBS) produces the intermediate, **A_32_**. Commercially available (*R*)-glycidyl benzyl ether (**A_33_**) induces chirality on aniline by acid-catalyzed ring opening, followed by reduction of the nitro group to give **A_34_**. Subsequent steps, including Sonogashira coupling, Larock-type cyclization, and acid-amine coupling, give tezacaftor in 84% yield.

#### 3.1.10. Pyrotinib Maleate (2018)

**Scheme 10:**
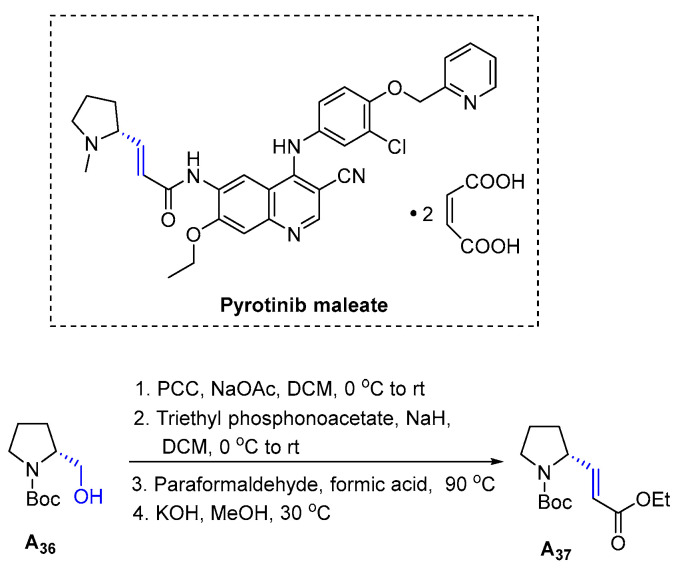
Synthesis of Pyrotinib maleate.

Hengrui Pharmaceuticals developed pyrotinib maleate [40], a pan-ErbB receptor tyrosine kinase inhibitor, which is used for the treatment of metastatic breast cancer. The kilogram-scale synthesis of pyrotinib maleate is shown in Scheme 10. Chiral fragment **A_37_** is synthesized from *N*-Boc-d-prolinol (**A_36_**) in four steps [41].

#### 3.1.11. Encorafenib (2018)

**Scheme 11:**
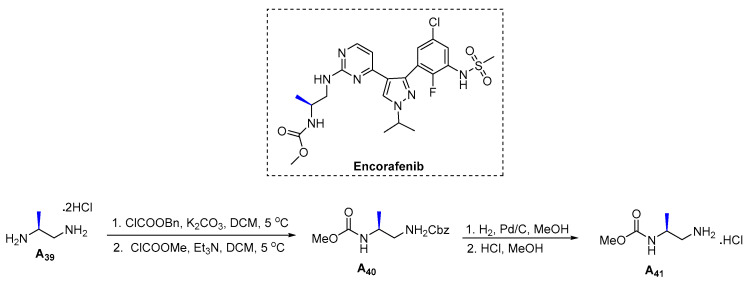
Synthesis of Encorafenib.

Encorafenib, in combination with binimetinib, is used for the treatment of metastatic melanoma [42]. Currently, encorafenib is marketed by Pfizer. Chirality is introduced as follows: (*S*)-(−)-1,2-diaminopropane dihydrochloride (**A_39_**) is treated with benzyl chloroformate and methyl chloroformate in the presence of a base, resulting in the carbamate derivative, **A_40_**. Upon hydrogenation, the chiral subunit, **A_41_**, is obtained (Scheme 11) [43].

#### 3.1.12. Duvelisib Monohydrate (2018)

**Scheme 12:**
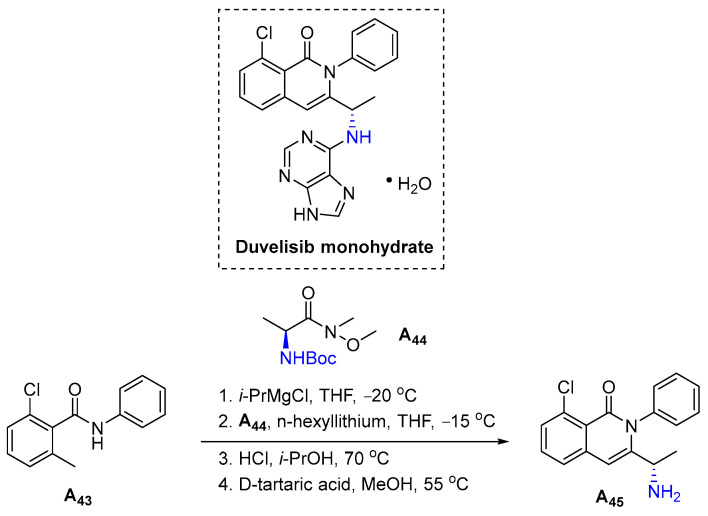
Synthesis of Duvelisib monohydrate.

Duvelisib monohydrate, initially developed by Intellikine and later licensed to Verastem Oncology [44], is used in the treatment of both chronic lymphocytic leukemia and small lymphocytic lymphoma [45,46]. The kilogram-scale synthesis of duvelisib disclosed by Intellikine is discussed in Scheme 12 [47]. Weinreb amide **A_44_** treated with n-hexyl lithium is combined with benzamide **A_43_** to form the chiral isoquinoline **A_45_** in four steps. In the last step, salt resolution is performed with d-tartaric acid in methanol, and then with ammonium hydroxide to enhance the enantiopurity of **A_45_**.

#### 3.1.13. Elagolix Sodium (2018)

**Scheme 13:**
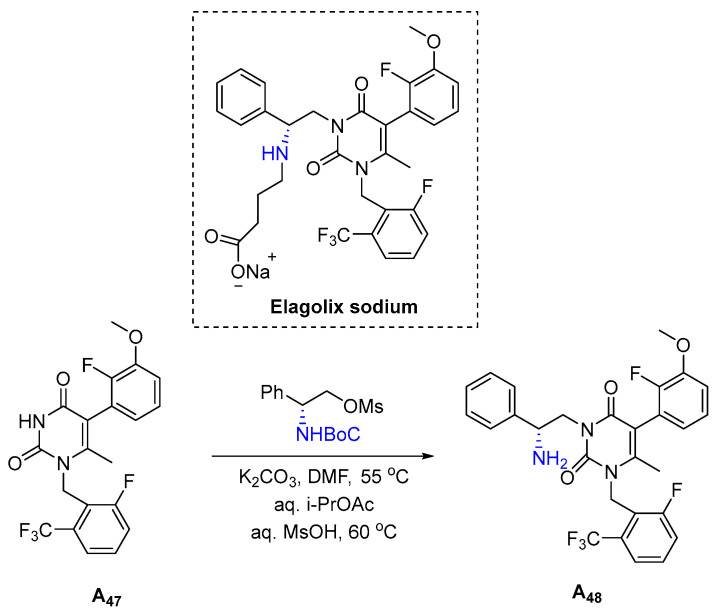
Synthesis of Elagolix sodium.

Elagolix sodium, used for treating women with endometriosis, was developed by Abbvie and Neurocrine Biosciences [48]. Similar to duvelisib monohydrate, chirality is induced during the alkylation of uracil with mesylate derivative; the latter compound is synthesized by the reaction of (−)-N-Boc-d-α-phenylglycinol with methanesulfonyl chloride (Scheme 13) [49].

#### 3.1.14. Tegoprazan (2018)

**Scheme 14:**
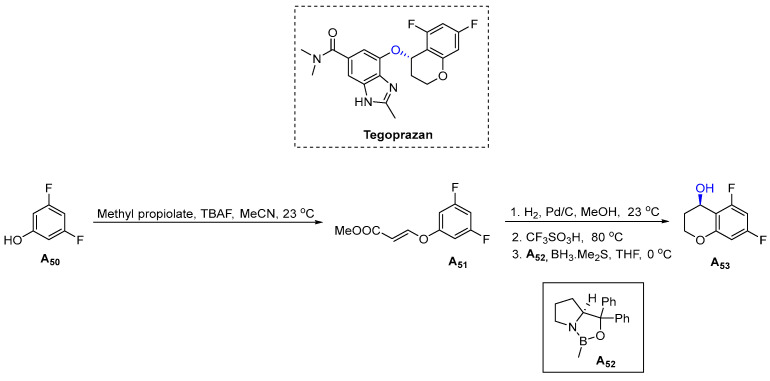
Synthesis of Tegoprazan.

Tegoprazan, initially discovered by Pfizer and further developed by RaQualia Pharmaceuticals and CJ Healthcare, is used for the treatment of gastroesophageal reflux disease (GERD) [50]. The scalable synthesis of tegoprazan is given in Scheme 14 [51]. The introduction of an enantiopure chromanol side chain **A_53_** on the benzimidazole ring is the key step of the synthesis that occurs in the presence of tri-*n*-butylphosphine with 1,1′-(azodicarbonyl)dipiperidine (ADDP). Then, 3,5-difluorophenol (**A_50_**) undergoes condensation with methyl propiolate to afford enol ethers (**A_51_**) in both the *E* and *Z* forms (1:1 mixture). Hydrogenation, followed by intramolecular Friedel–Crafts acylation and asymmetric reduction with ozaborolidine catalyst (**A_52_**), produces chromanol (**A_53_**). Finally, recrystallization of the latter compound yields the enantiopure chiral subunit, **A_53_**.

#### 3.1.15. Alpelisib (2019)

**Scheme 15:**
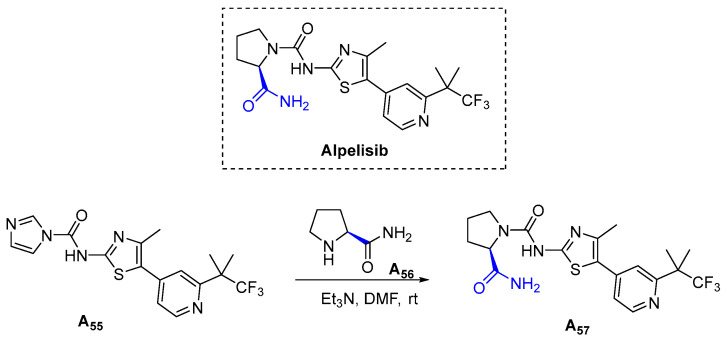
Synthesis of Alpelisib.

Novartis Pharmaceuticals developed alpelisib [52,53]. It is used for the treatment of metastatic breast cancer. The chiral center is introduced by l-proline amide **A_56_** on the imidazole ring of **A_55_** in the presence of triethylamine during the final step of synthesis (Scheme 15) [54,55,56].

#### 3.1.16. Solriamfetol (2019)

**Scheme 16:**
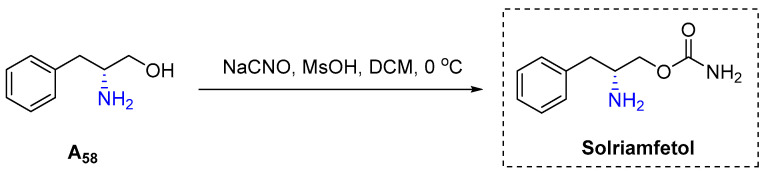
Synthesis of Solriamfetol.

Solriamfetol, developed by SK Biopharmaceuticals [57], is used for the treatment of Ehlers–Danlos syndromes (EDS) associated with obstructive sleep apnea (OSA) and narcolepsy [58]. The kilogram-scale synthesis of solriamfetol is shown in Scheme 16 [59]. Solriamfetol is synthesized in 89% yield in a single step from d-phenylalaninol (**A_58_**) and sodium cyanate in the presence of acid.

#### 3.1.17. Pretomanid (2019)

**Scheme 17:**
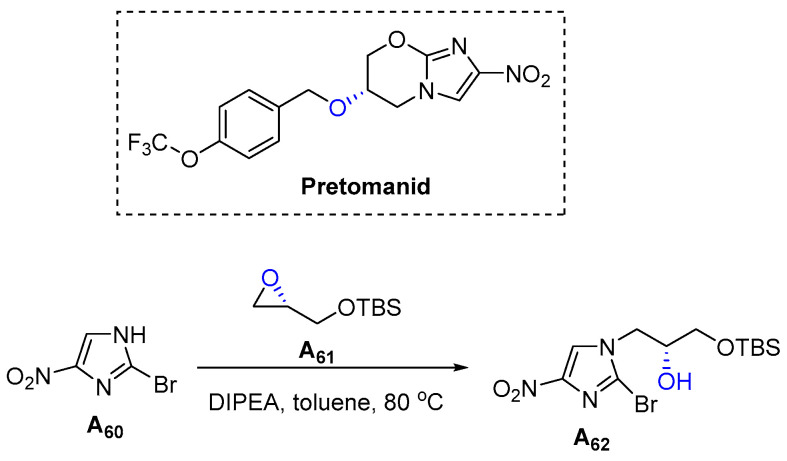
Synthesis of Pretomanid.

Pretomanid, an antimycobacterial agent, is used to treat tuberculosis (TB). It is the first TB drug to be developed by TB Alliance [60]. Epoxide (**A_61_**) plays its role as a chiral substrate by coupling with prochiral nitroimidazole (**A_60_**) in the presence of DIPEA, affording **A_62_** (Scheme 17) [61].

#### 3.1.18. Zanubrutinib (2019)

**Scheme 18:**
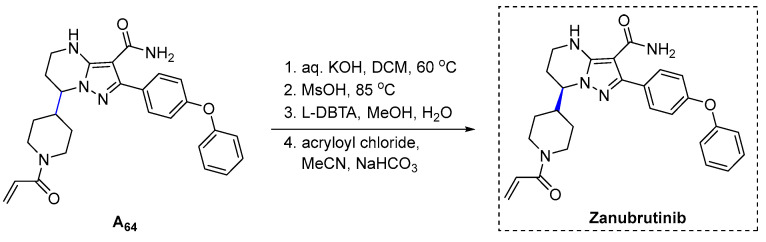
Synthesis of Zanubrutinib.

BeiGene, Inc. (Cambridge, MA, USA) developed zanubrutinib for the treatment of mantle cell lymphoma [62]. In contrast to the chiral pool approach discussed above, Zanubrutinib is the first drug that is produced by chiral resolution. Zanubrutinib is obtained in its pure enantiomeric form via resolution methodology by treating it with l-dibenzoyltartaric acid (LDBTA) (Scheme 18) [63].

#### 3.1.19. Darolutamide (2019)

**Scheme 19:**
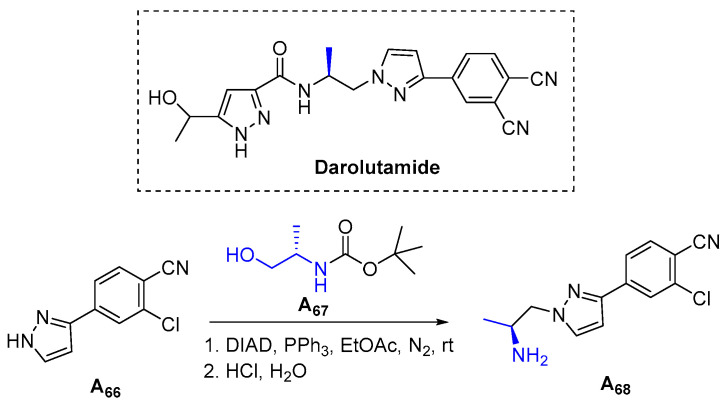
Synthesis of Darolutamide.

Darolutamide is used in the treatment of non-metastatic castrate-resistant prostate cancer [64]. It was developed by Orion Corporation and Bayer Healthcare [65]. The key step in the asymmetric synthesis of darolutamide is the insertion of chiral isopropylamine fragment, **A_67_**, into the biaryl scaffold in **A_66_**, followed by acid-mediated deprotection of the Boc group to generate an intermediate **A_68_** (Scheme 19) [66].

#### 3.1.20. Cenobamate (2019)

**Scheme 20:**
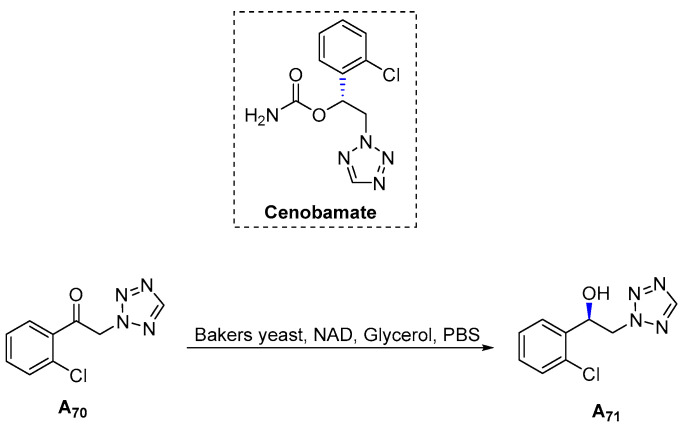
Synthesis of Cenobamate.

SK Pharmaceuticals developed cenobamate [67] for the treatment of partial-onset seizures. The enzymatic catalysis presented in Scheme 20 [68] outlines its asymmetric catalytic hydrogenation in the presence of *Rhodotorula mucilaginosa*, an oxidoreductase.

#### 3.1.21. Avapritinib (2020)

**Scheme 21:**
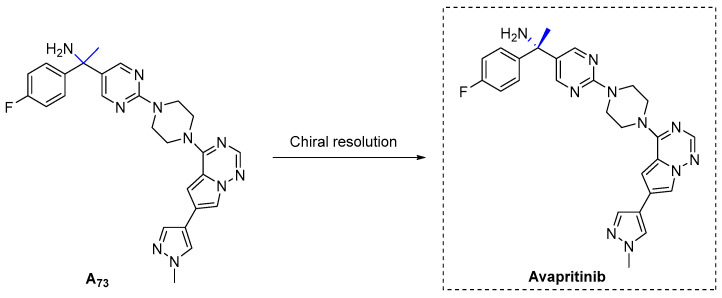
Synthesis of Avapritinib.

Blueprint Medicines developed avapritinib [69] for the treatment of metastatic gastrointestinal tract cancers. The chiral resolution by means of supercritical fluid chromatographic separation of intermediate, **A_73_**, produces enantiopure avapritinib in 68% yield (Scheme 21) [70].

#### 3.1.22. Berotralstat (2020)

**Scheme 22:**
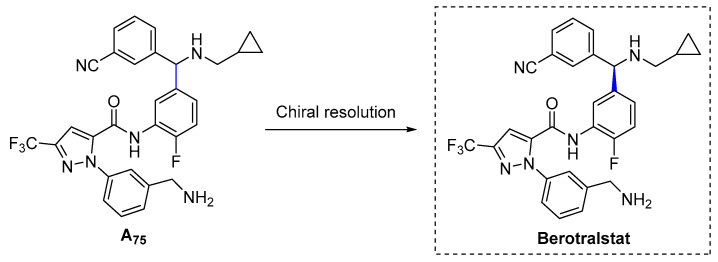
Synthesis of Berotralstat.

Berotralstat is used to treat the prophylaxis of hereditary angioedema (HAE) attacks [71]. Following the same procedure as avapritinib, berotralstat is synthesized in its pure enantiomeric form by means of supercritical fluid chromatography in the final step (Scheme 22) [72].

#### 3.1.23. Lonafarnib (2020)

**Scheme 23:**
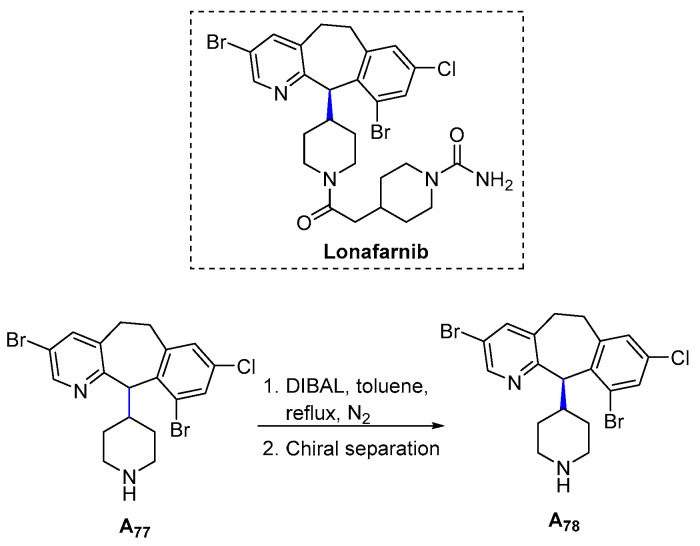
Synthesis of Lonafarnib.

Lonafarnib is used for the treatment of Hutchinson–Gilford progeria syndrome (HGPS) [73]. Unlike berotralstat, chirality in lonafarnib is obtained by chiral separation. Intermediate **A_77_** upon reduction with diisobutylaluminium hydride (DIBAL) followed by chiral separation resulted in the formation of the chiral subunit, **A_78_**, with one stereocenter. These successive steps yield lonafarnib (Scheme 23) [74].

#### 3.1.24. Osilodrostat (2020)

**Scheme 24:**
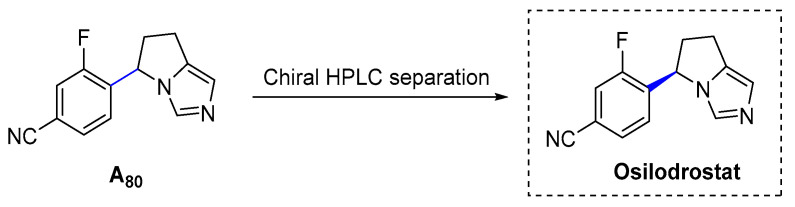
Synthesis of Osilodrostat.

Osilodrostat, used for treating Cushing’s disease, is a 11*β*-hydroxylase inhibitor [75]. Chiral osilodrostat is generated in a manner similar to that of berotralstat. Chiral HPLC separation of the intermediate **A_80_** directly results in drug formation (Scheme 24) [76].

#### 3.1.25. Oliceridine (2020)

**Scheme 25:**
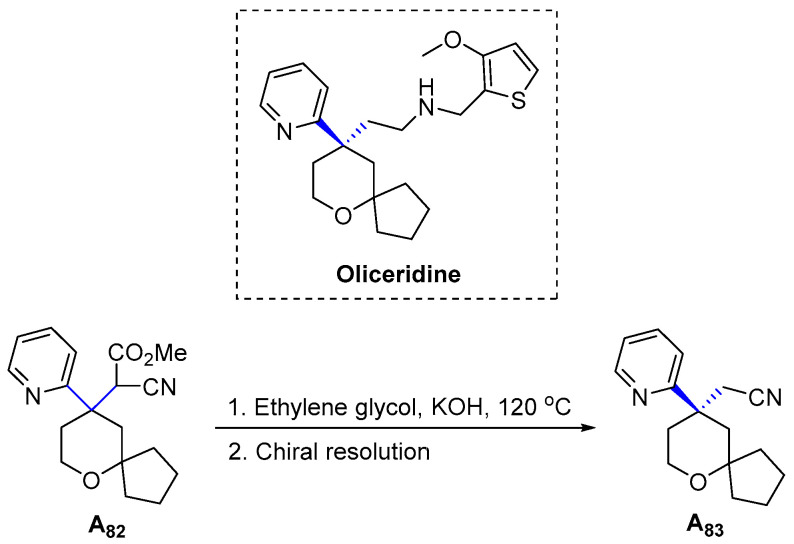
Synthesis of Oliceridine.

Oliceridine, a central nervous system (CNS) drug, is used to treat moderate-to-severe acute pain [77]. In the total synthesis of oliceridine, the chiral intermediate **A_83_** is obtained by the decarboxylic reaction of **A_82_**, followed by SFC chiral separation (Scheme 25) [78].

#### 3.1.26. Remimazolam (2020)

**Scheme 26:**
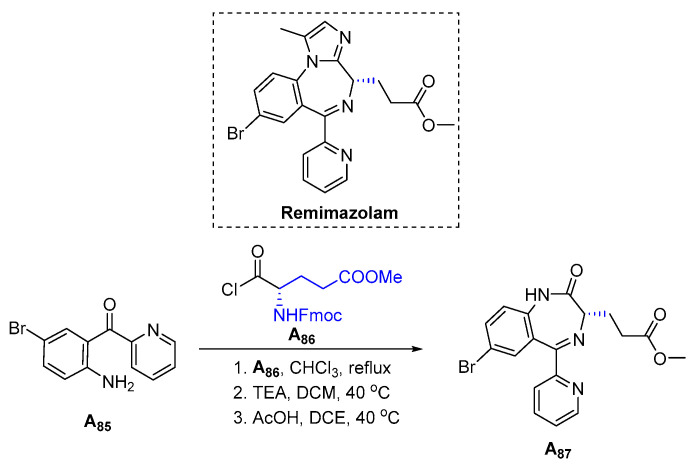
Synthesis of Remimazolam.

Acacia Pharma developed remimazolam, an ultrashort-acting benzodiazepine [79]. Treatment of **A_85_** with the chiral substrate, **A_86_** in chloroform generated the substituted product, which upon base-promoted F-moc deprotection and acetic acid promoted condensation resulted in the formation of cyclized intermediate **A_87_** in three steps (Scheme 26) [80].

#### 3.1.27. Ozanimod (2020)

**Scheme 27:**
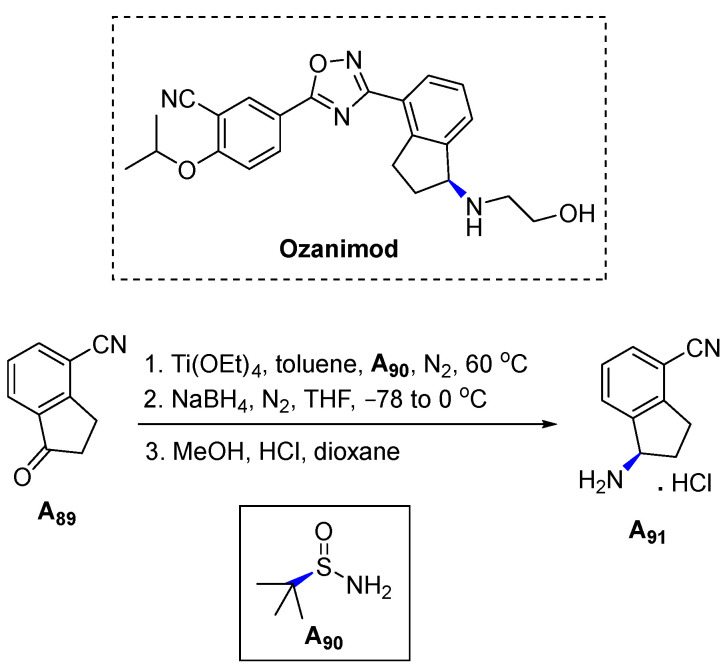
Synthesis of Ozanimod.

Ozanimod is used to treat relapsing multiple sclerosis [81]. The chiral sulfinamide fragment, **A_90_**, acts as a chiral auxiliary group by inserting a chiral center on the indene derivative, **A_89_**. Ozanimod is synthesized in successive steps from intermediate **A_91_** (Scheme 27) [82].

### 3.2. Drugs with Two Chiral Centers

#### 3.2.1. Nemonoxacin (2016)

**Scheme 28:**
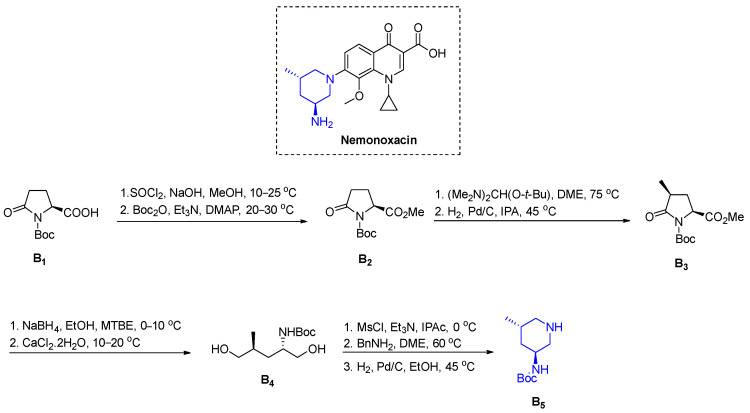
Synthesis of Nemonoxacin.

Nemonoxacin was originally developed by Procter & Gamble Pharmaceuticals [83] (P&GP) and co-developed by TaiGen Biotechnology (Asia) and Warner Chilcott (US and Europe). Scheme 28 describes the process-scale synthesis of nemonoxacin [84]. Proline derivative **B_1_**, upon esterification, gives an intermediate (**B_2_**), which, on treatment with Bredereck’s reagent and hydrogenation, gives chiral subunit **B_3_**. Then, simultaneous reduction and treatment with CaCl_2_ form the diol **B_4_**. Aminopiperidine **B_5_** is obtained from this intermediate via sequential mesylation, cyclization, and hydrogenation.

#### 3.2.2. Brivaracetam (2016)

**Scheme 29:**
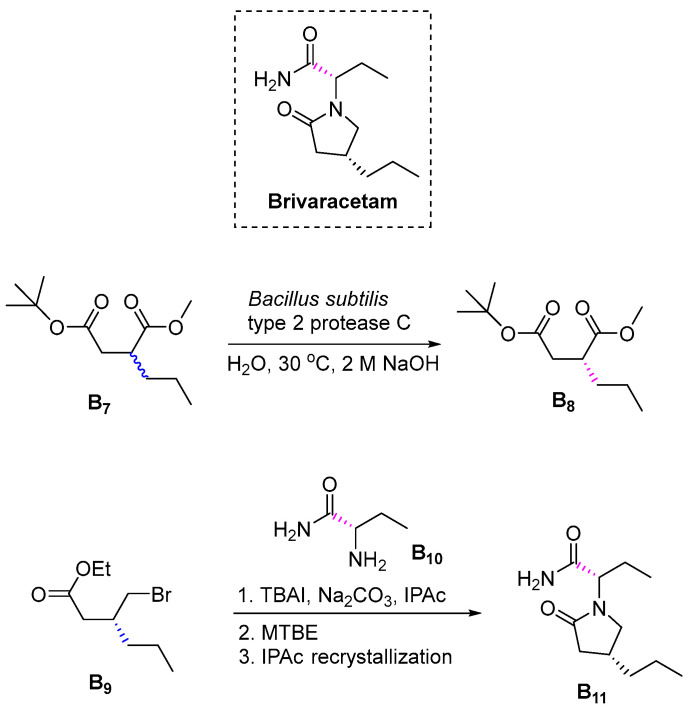
Synthesis of Brivaracetam.

UCB Pharma developed both brivaracetam and levetiracetam. Brivaracetam is an antiepileptic drug used to treat partial-onset seizures [85,86]. The kilogram-scale synthesis shown in Scheme 29 [87] shows that chirality is achieved by enzymatic resolution with protease C to form fragment **B_8_**. Another chiral pool substrate, (*S*)-2-aminobutanamide (**B_10_**), is inserted into the chiral subunit, **B_9_** to form brivaracetam **B_11_**.

#### 3.2.3. Beclabuvir (2016)

**Scheme 30:**
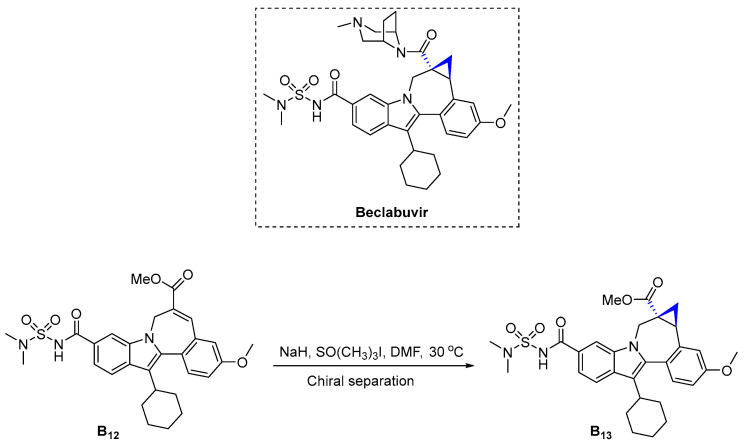
Synthesis of Beclabuvir.

Bristol Myers Squibb discovered and developed beclabuvir [88]. It is used to treat HCV infections. The chiral cyclopropyl fragment, B_13_, is generated by the Corey–Chaykovsky reaction using NaH, followed by chiral separation. Beclabuvir is synthesized from intermediate B_13_ in successive steps (Scheme 30) [89].

#### 3.2.4. Inotuzumab Ozogamicin (2017)

**Scheme 31:**
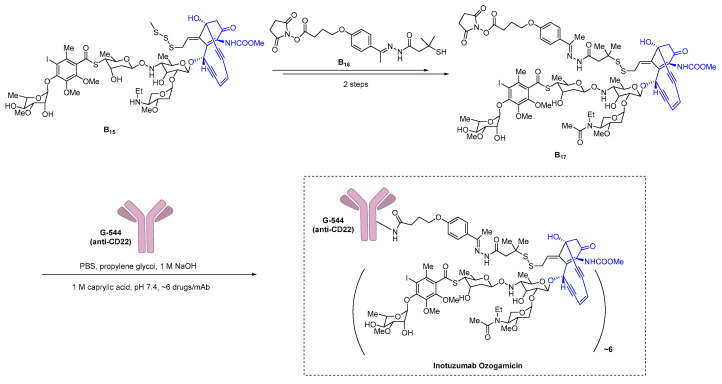
Synthesis of Inotuzumab Ozogamicin.

Inotuzumab Ozogamicin, discovered by Lederle Laboratories, is used for the treatment of refractory B-cell precursor acute lymphoblastic leukemia (ALL) [90]. Scheme 31 provides an overview of the chiral pool synthesis of inotuzumab ozogamicin. Fermentation of *Micromonospora echinospora* sp. *Calichenis* gave Calicheamicin (**B_15_**) [91,92]. The linker (**B_16_**) is then coupled to chiral substrate B_15_ to produce **B_17_**. Later, it is conjugated with anti-CD22 mAb G-544 to obtain inotuzumab ozogamicin in 60% yield [93].

#### 3.2.5. Deutetrabenazine (2017)

**Scheme 32:**
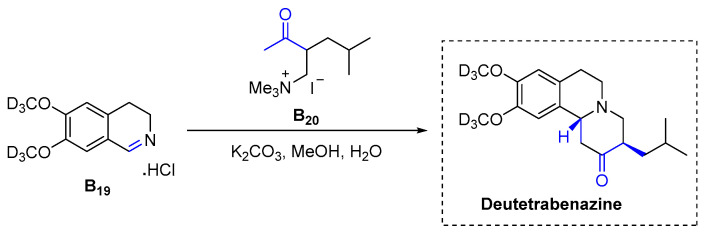
Synthesis of Deutetrabenazine.

Deutetrabenazine has been approved for the treatment of chorea (abnormal involuntary movements) associated with Huntington’s disease [94]. The synthesis of deutetrabenazine is described in Scheme 32 [95]. The reaction of dihydroisoquinoline fragment **B_19_** and quaternary ammonium salt **B_20_** in the presence of a base resulted in the formation of the desired product. Unfortunately, deutetrabenazine is obtained as a racemic mixture (cis-diastereomer) in 67% yield.

#### 3.2.6. Vaborbactam (2017)

**Scheme 33:**
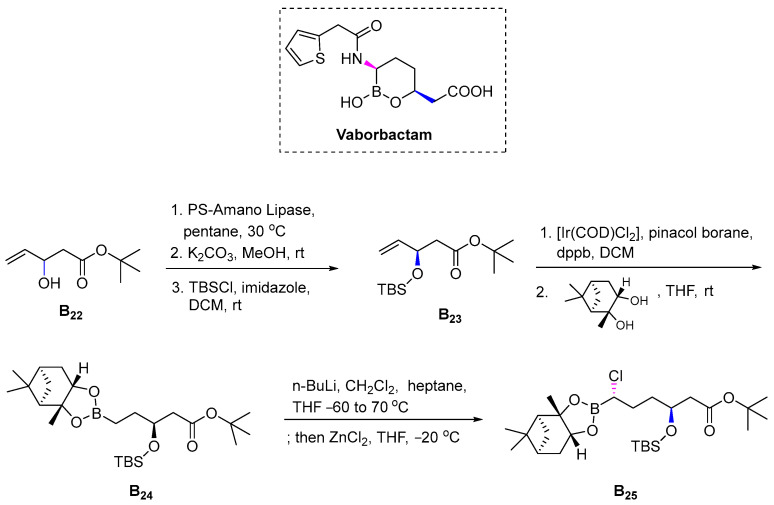
Synthesis of Vaborbactam.

Rempex Pharmaceuticals discovered vaborbactam, and it was then developed by The Medicines company. Vaborbactam, in combination with meropenem, is used to treat complicated urinary tract infections [96]. The key step in its synthesis is obtaining a pure enantiomeric form by enantioselective lipase resolution of the racemic substrate, **B_22_**. Subsequently, the chiral centers are inserted via Matteson homologation (Scheme 33) [97].

#### 3.2.7. Telotristat Ethyl (2017)

**Scheme 34:**
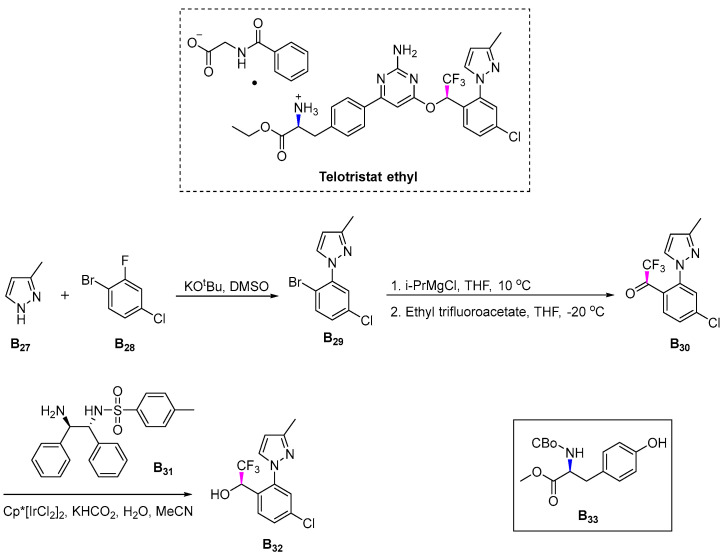
Synthesis of Telotristat ethyl.

Lexicon Pharmaceuticals developed telotristat ethyl, which has two chiral carbons, for the treatment of carcinoid syndrome diarrhea [98]. One of the chiral carbons is implanted from commercially available N-Boc-tyrosine methyl ester **B_33_** and the other, shown in **B_32_**, from asymmetric transfer hydrogenation using an Iridium catalyst and ligand **B_31_** (Scheme 34) [99,100]. 

#### 3.2.8. Larotrectinib (2018)

**Scheme 35:**
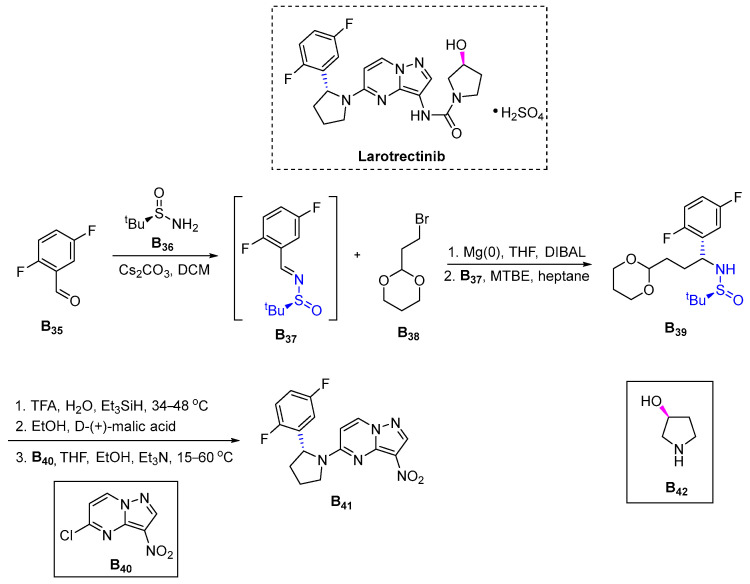
Synthesis of Larotrectinib.

Array BioPharma and LOxo Oncology discovered larotrectinib, which was further developed in collaboration with Bayer AG. It is used to treat solid tumors with neurotrophic receptor tyrosine kinase gene fusions [101]. Ellman’s auxiliary (**B_36_**) is responsible for the chirality in larotrectinib. The second stereogenic center is derived from a commercially available pyrrolidinol fragment (Scheme 35) [102].

#### 3.2.9. Glasdegib (2018)

**Scheme 36:**
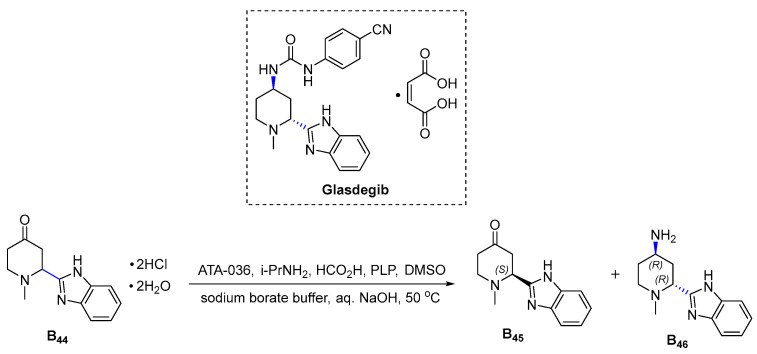
Synthesis of Glasdegib.

Glasdegib was developed by Pfizer and is used for the treatment of acute myeloid leukemia [103]. Glasdegib is a good example of chiral resolution in drug synthesis. The key chiral substrate, **B_46_**, (anti-form) is obtained by dynamic kinetic resolution of the racemic mixture, **B_44_**, with transaminase enzyme ATA-306 in the presence of borate buffer (Scheme 36) [104].

#### 3.2.10. Talazoparib (2018)

**Scheme 37:**
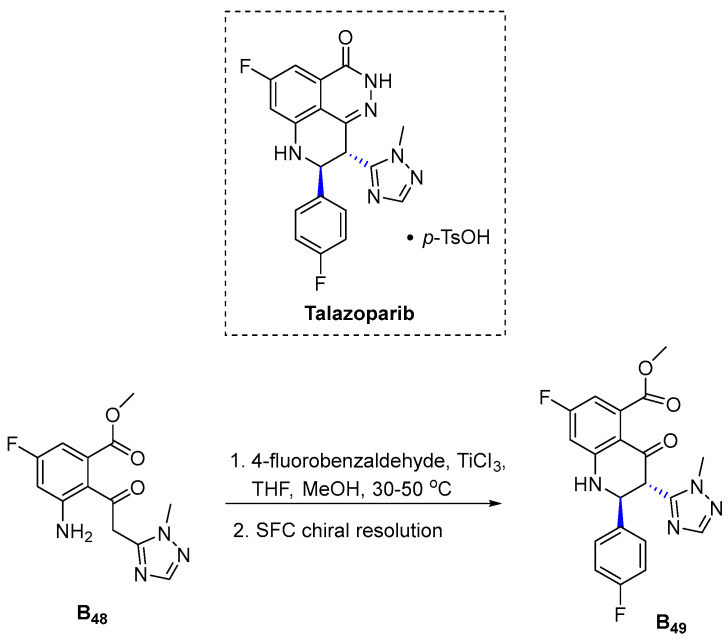
Synthesis of Talazoparib.

Talazoparib, discovered by BioMarin and developed by Pfizer, is used to treat germline BRCA-mutated HER2-negative metastatic breast cancer [105]. Unlike glasdegib, the synthesis of talazoparib employs supercritical fluid chromatography (SFC) chiral separation to obtain the essential 1,2,4-triazole subunit, **B_49_**, which has two chiral centers (Scheme 37) [106,107,108].

#### 3.2.11. Ivosidenib (2018)

**Scheme 38:**
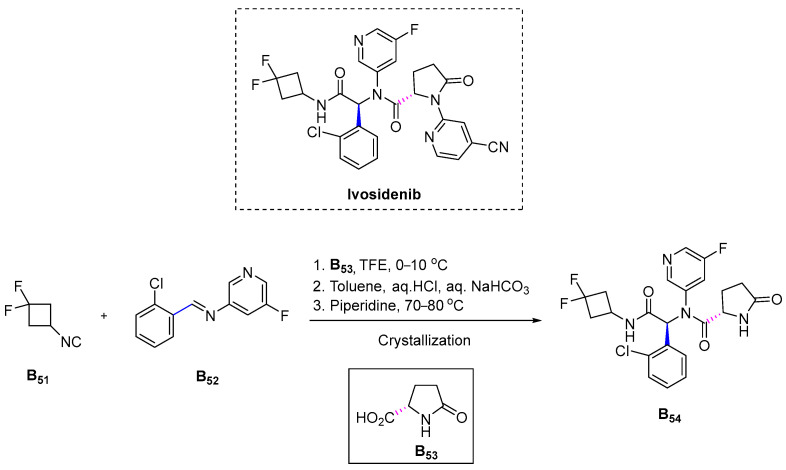
Synthesis of Ivosidenib.

Agios Pharmaceuticals developed ivosidenib for the treatment of relapsed or refractory acute myeloid leukemia [109]. The Ugi reaction between isonitrile (**B_51_**), imine (**B_52_**), and chiral acid (**B_53_**) results in a racemic intermediate. Crystallization, followed by piperidine treatment, affords diastereomer **B_54_**. The crystallization step is considered crucial because the final synthesis of the drug relies on the diastereomer alone (Scheme 38) [110].

#### 3.2.12. Evocalcet (2018)

**Scheme 39:**
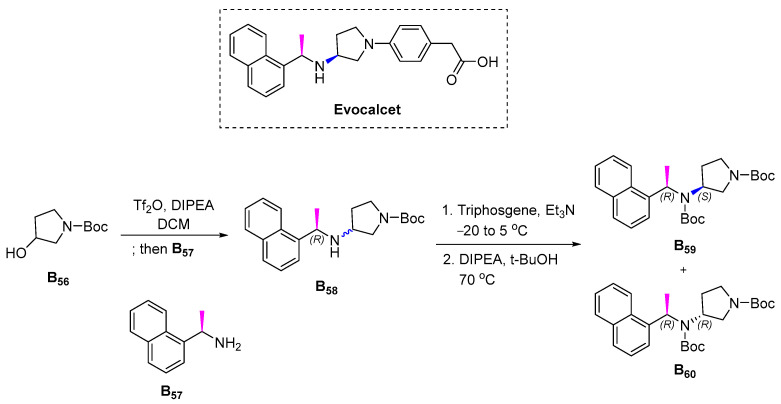
Synthesis of Evocalcet.

The Mitsubishi Tanabe Pharma corporation discovered evocalcet and Kyowa Kirin further developed the drug for secondary hyperparathyroidism (SHPT) treatment [111]. The diastereomeric mixture, **B_58_**, obtained from N-Boc pyrrolidinol (**B_56_**), is treated with triphosgene and tert-butanol to obtain both syn (**B_59_**) and anti (**B_60_**) diastereomers. These two compounds are separated by chromatography. For this drug, the chromatography approach is predominantly used over the chiral pool approach, as evocalcet, with two chiral centers, is synthesized from the syn-pyrrolidine derivative, **B_59_** (Scheme 39) [111].

#### 3.2.13. Baloxavir Marboxil (2018)

**Scheme 40:**
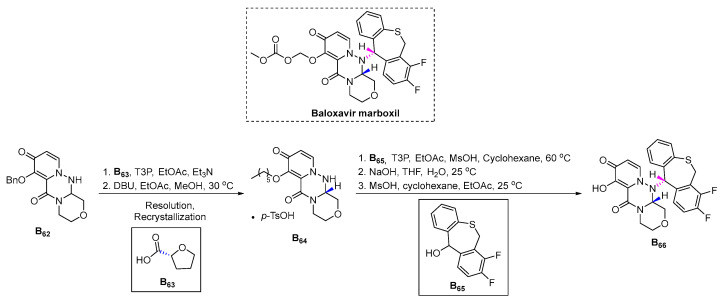
Synthesis of Baloxavir marboxil.

Baloxavir marboxil was first approved by Pharmaceuticals and Medical Devices Agency (PMDA) and was further developed by Shionogi Inc. for the treatment of influenza A and B infections [112]. Baloxavir marboxil is constructed from different subunits, including chiral piperazine (**B_64_**), benzothiepine (**B_65_**), and alkyl chloride side chains. Resolution is carried out via the reaction of piperazine fragment **B_62_** with chiral acid **B_63_**. Subsequent recrystallization results in **B_64_** (Scheme 40) [113].

#### 3.2.14. Fosravuconazole l-Lysine Ethanolate (2018)

**Scheme 41:**
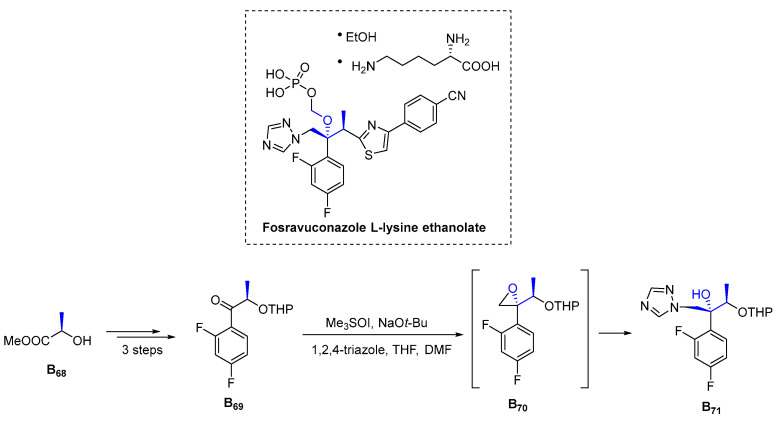
Synthesis of Fosravuconazole l-lysin ethanolate.

Eisai Co., Ltd. (Bunkyo City, Tokyo) discovered fosravuconazole l-lysine ethanolate [114], a prodrug of ravuconazole and broad-spectrum antifungal agent. The process-scale synthesis of fosravuconazole is outlined in Scheme 41 [115]. The chiral pool synthesis of fosravuconazole l-lysine ethanolate starts with a single chiral center in lactate (**B_68_**) and another is induced from intermediate **B_69_** by Corey–Chaykovsky epoxidation and sequential ring opening in a single step. Ravuconazole is converted into fosravuconazole in three successive steps (Scheme 41) [116].

#### 3.2.15. Lumateperone (2019)

**Scheme 42:**
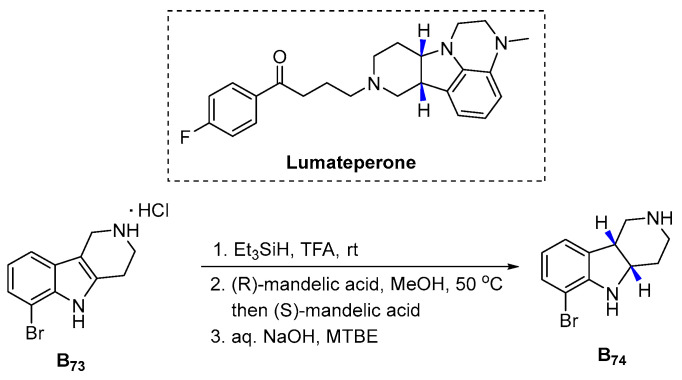
Synthesis of Lumateperone.

Lumateperone is an antipsychotic drug used to treat schizophrenia [117]. In the nine-step synthesis of lumateperone, stereocenters are generated during the reduction of tricyclic indole intermediate **B_73_** with triethylsilyl hydride, followed by treatment with (*R*)-mandelic acid in methanol. Thus, the formed (*S*)-mandelic acid diastereomeric salt undergoes free-basing with aqueous NaOH to afford chirally pure cis-indoline **B_74_** (Scheme 42) [118].

#### 3.2.16. Tenapanor (2019)

**Scheme 43:**
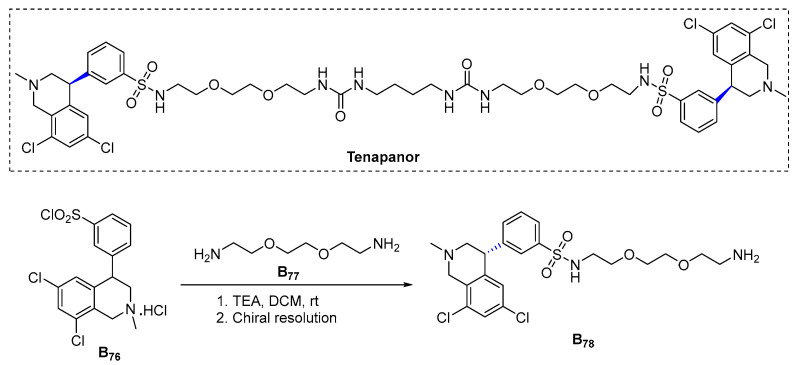
Synthesis of Tenapanor.

Tenapanor was developed by Ardelyx Inc. to treat irritable bowel syndrome with constipation [119]. Scheme 43 describes the synthesis of tenapanor. The tetrahydroisoquinoline intermediate, **B_76_**, generated during the synthesis reacts with linker **B_77_**, followed by chiral resolution (SFC separation), resulting in the formation of chiral fragment **B_78_** [120].

#### 3.2.17. Lemborexant (2019)

**Scheme 44:**
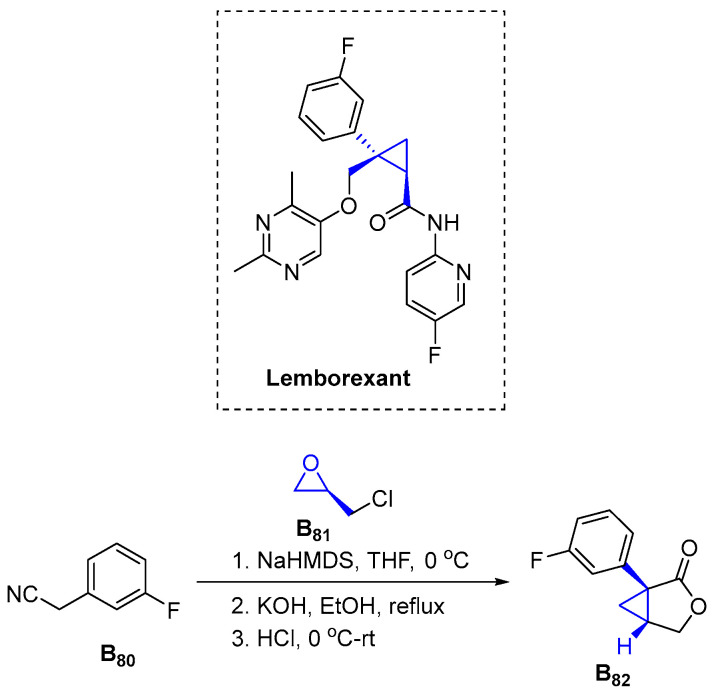
Synthesis of Lemborexant.

Lemborexant, developed by Eisai Co., Ltd. is used for the treatment of insomnia [121]. Starting with 2-(3-fluorophenyl) acetonitrile (**B_80_**), chirality is induced from epoxide **B_81_** by step-by-step substitution, hydrolysis, ring-opening, and ring-closure reactions. Thus, the generated intermediate **B_82_** undergoes reduction with NaBH_4_, followed by lipase-induced transesterification, eventually leading to the synthesis of lemborexant in five steps (Scheme 44) [122].

#### 3.2.18. Cefiderocol (2019)

**Scheme 45:**
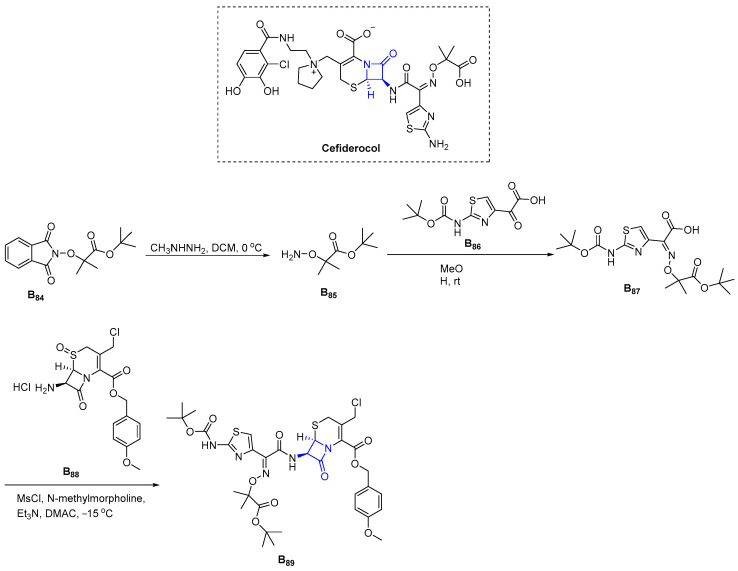
Synthesis of Cefiderocol.

Shionogi et al. developed cefiderocol, a cephalosporin antibacterial drug. It is used for the treatment of complicated urinary tract infections (cUTI) [123]. Linear synthesis of cefiderocol is shown in Scheme 45 [124]. Chirality is induced by azetidinone ring **B_88_**, which was previously synthesized from the phthalimide derivative, **B_84_**.

#### 3.2.19. Upadacitinib (2019)

**Scheme 46:**
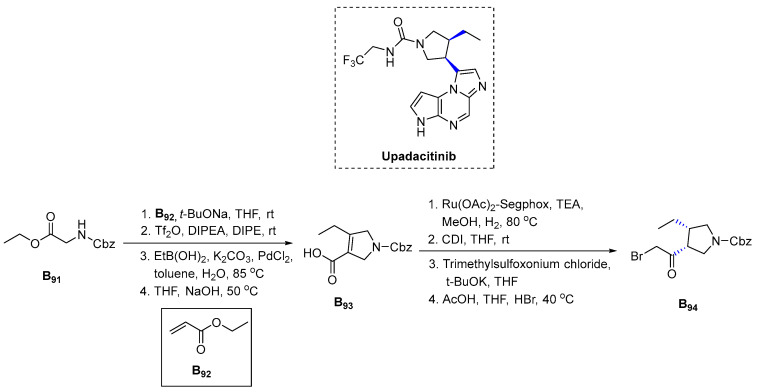
Synthesis of Upadacitinib.

Upadacitinib is used to treat rheumatoid arthritis [125]. Chiral pyrrolidine fragment **B_94_**, which is essential for the synthesis of upadacitinib, is obtained by asymmetric hydrogenation of **B_93_** in the presence of a ruthenium catalyst. The latter compound **B_93_** was obtained by the condensation of Cbz-protected glycine ethyl ester **B_91_** and ethyl acrylate **B_92_** in the presence of sodium tertiary butoxide (Scheme 46) [126].

### 3.3. Drugs with Three Chiral Centers

#### 3.3.1. Tenofovir Alafenamide Fumarate (2016)

**Scheme 47:**
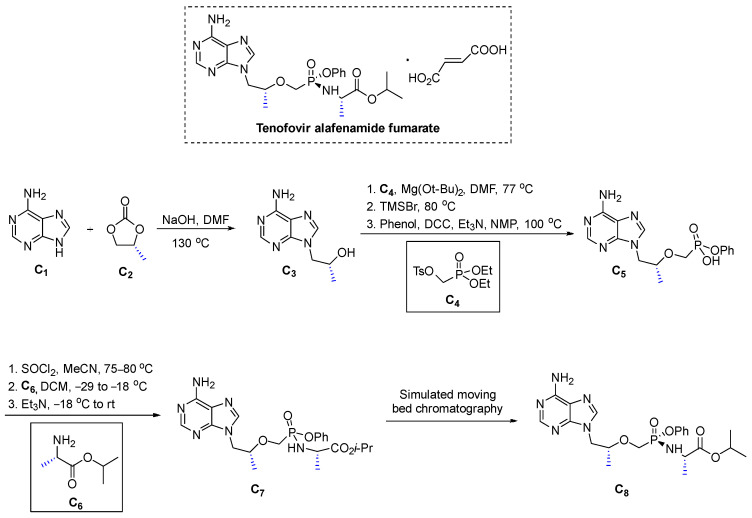
Synthesis of Tenofovir alafenamide fumarate.

Tenofovir alafenamide fumarate was discovered and developed by Gilead for the treatment of chronic hepatitis B viral infections [127]. The reaction of adenine (**C_1_**) with (R)-propylene carbonate (**C_2_**) affords intermediate **C_3_**, with a single chiral center. In successive steps, the monophosphonate ester, **C_5_**, is treated with thionyl chloride, and the l-alanine derivative, **C_6_**, affords racemic intermediate **C_7_**, with two chiral centers. Simulated moving bed chromatography is employed to resolve the racemic mixture and obtain diastereomers. Tenofovir alafenamide fumarate is synthesized from diastereomer **C_8_** and fumaric acid (Scheme 47) [128].

#### 3.3.2. Bictegravir (2018)

**Scheme 48:**
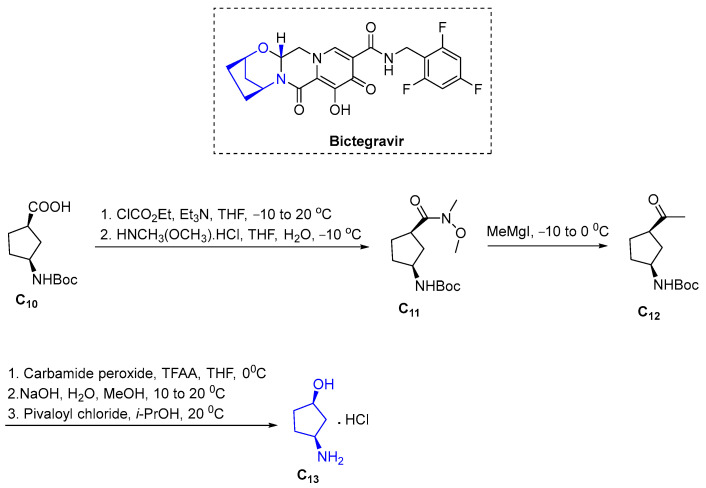
Synthesis of Bictegravir.

A combination of bictegravir, emtricitabine, and tenofovir alafenamidecan be used to treat HIV-1 infections [129]. The process-scale synthesis of bictegravir was reported and developed by Gilead (Scheme 48) [130,131]. The late-stage installation of chiral substrate **C_13_** to the pyridone derivative results in bictegravir in three steps. Synamino pentanol (**C_13_**) is previously obtained from commercially available cyclopentanoic acid (**C_10_**) in six steps. Acid **C_10_** is converted to amide **C_11_**, which then undergoes oxidation to form the chiral alcohol subunit, **C_13_**.

#### 3.3.3. Relebactam (2019)

**Scheme 49:**
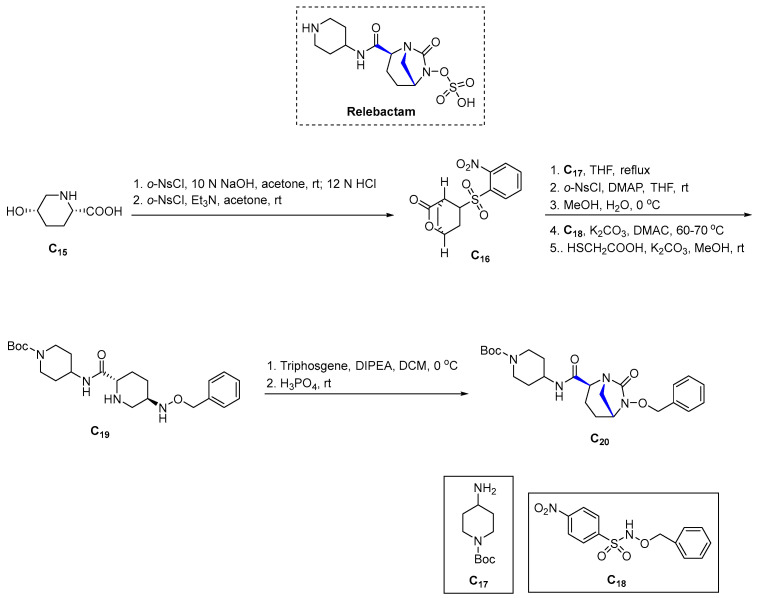
Synthesis of Relebactam.

Relebactam was developed by Merk Sharp & Dohme [132]. Relebactam, imipenem, and cilastin is a drug combination used for the treatment of complicated urinary tract and intra-abdominal infections [133]. Chiral substrate 5-hydroxypiperidine-2-carboxylic acid (**C_15_**) undergoes sulfonylation and intramolecular esterification to form the bridged intermediate, **C_16_**. Later, ring opening followed by substitution and cyclization with triphosgene gives the chiral bridged intermediate, **C_20_**, with three chiral centers (Scheme 49) [134].

#### 3.3.4. Fam-Trastuzumab Deruxtecan-Nxki (2019)

**Scheme 50:**
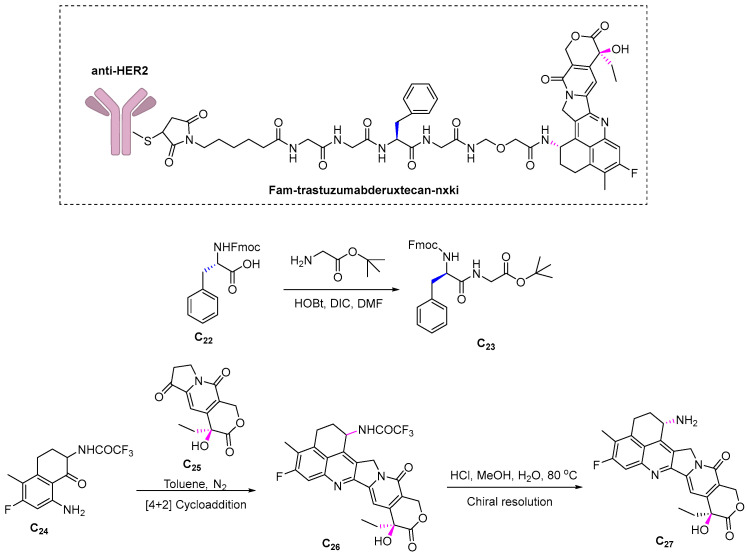
Synthesis of Fam-trastuzumabderuxtecan-nxki.

Daiichi Sankyo and AstraZeneca developed fam-trastuzumab deruxtecan-nxki, an antibody–drug conjugate. The structure is composed of the GGFG linker **C_23_**, polycyclic chiral fragment **C_27_**, and anti-HER2 monoclonal antibody (mAb). Various stages of inducing chirality in a single drug are shown in Scheme 50 and described below [135]:

1. The linker **C_23_** with one chiral center is obtained by a chiral pool approach (i.e., amino acid derivative **C_22_** imparts its stereocenter to the drug);
2. The polycyclic chiral fragment **C_27_** is prepared by [4 + 2] cycloaddition, followed by chiral resolution adopting supercritical fluid chromatography of the racemic intermediate, **C_26_**.

#### 3.3.5. Pralsetinib (2020)

**Scheme 51:**
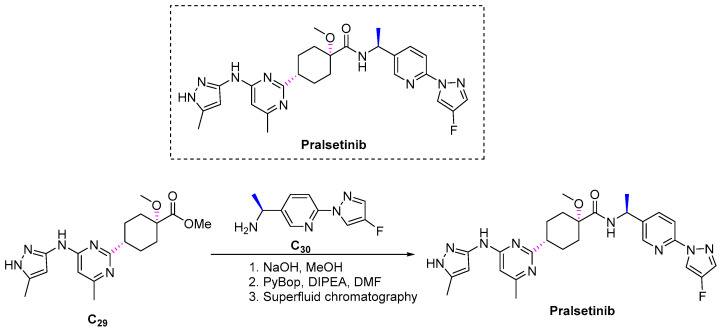
Synthesis of Pralsetinib.

Pralsetinib, developed by Blueprint Medicines, is used in the treatment of metastatic non-small cell lung cancer [136]. The key step during its synthesis is the benzotriazole-1-yl-oxytripyrrolidinophosphonium hexafluorophosphate (PyBOP)-stimulated amide coupling between the ester intermediate **C_29_** and the chiral amine fragment **C_30_**, to result in a mixture of diastereomers. The last step involves the isolation of pralsetinib by superfluid chromatography (Scheme 51) [137].

#### 3.3.6. Rimegepant (2020)

**Scheme 52:**
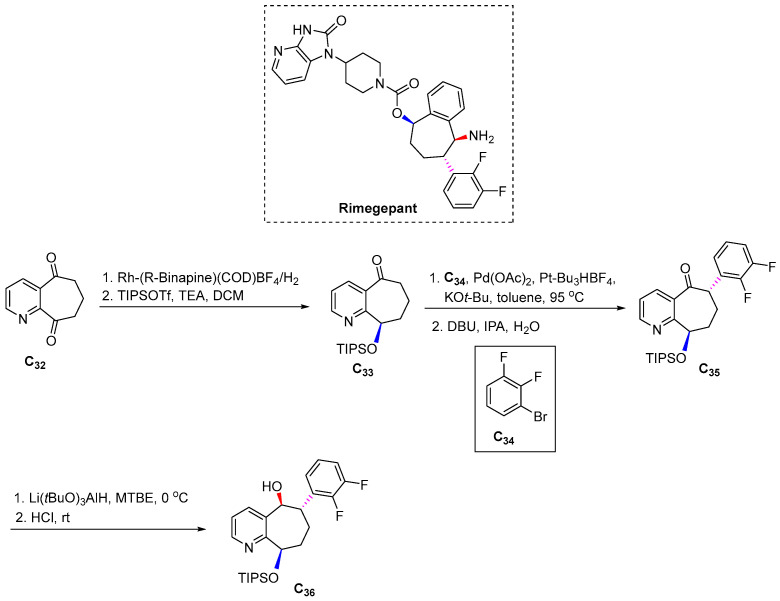
Synthesis of Rimegepant.

Biohaven developed rimegepant for the treatment of migraines [138]. The asymmetric synthesis of rimegepant is carried out by the following step-by-step process (Scheme 52) [139]:

1. Rhodium-catalyzed reduction of pyridine derivative **C_32_** followed by hydroxyl protection with triisopropylsilyl trifluoromethanesulfonate (TIPSOTf) results in a chiral intermediate **C_33_**;
2. Chiral fragment **C_33_** then undergoes coupling with 1-bromo-2,3-difluorobenzene **C_34_** resulting in an intermediate **C_35_** with two stereocenters;
3. The last chiral center in **C_36_** is obtained by lithium-mediated reduction.

### 3.4. Drugs with Four Chiral Centers

#### 3.4.1. Migalastat Hydrochloride (2016)

**Scheme 53:**
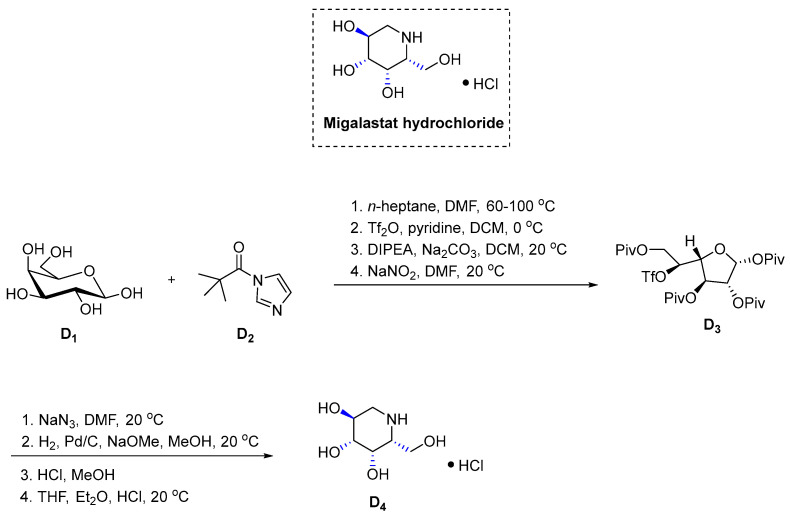
Synthesis of Migalastat hydrochloride.

Migalastat, also known as d-1-deoxygalactonojirimycin, is used to treat Fabry disease [140]. The kilogram-scale synthesis depicted in Scheme 53 [141] provides a clear example of the chiral pool synthesis of migalastat hydrochloride (**D_4_**) from d-galactose (**D_1_**).

#### 3.4.2. Midostaurin (2017)

**Scheme 54:**
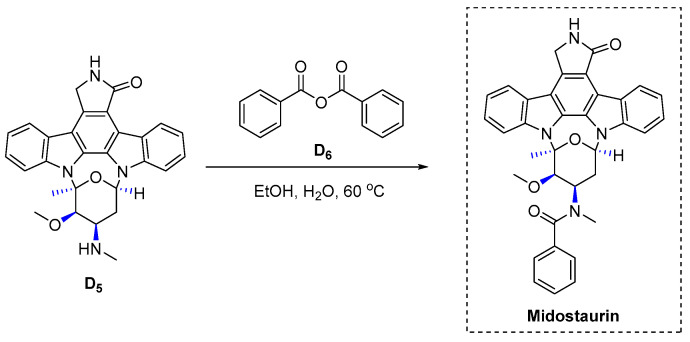
Synthesis of Midostaurin.

Midostaurin is used for the treatment of FLT3 mutation-positive Acute Myeloid Leukemia (AML) [142]. Its chiral pool synthesis begins with staurosporine (**D_5_**), a molecule produced by fermentation. Acylation of chiral substrate **D_5_** with benzoic anhydride (**D_6_**) results in the formation of midostaurin in a single step (Scheme 54) [143].

#### 3.4.3. Naldemedine (2017)

**Scheme 55:**
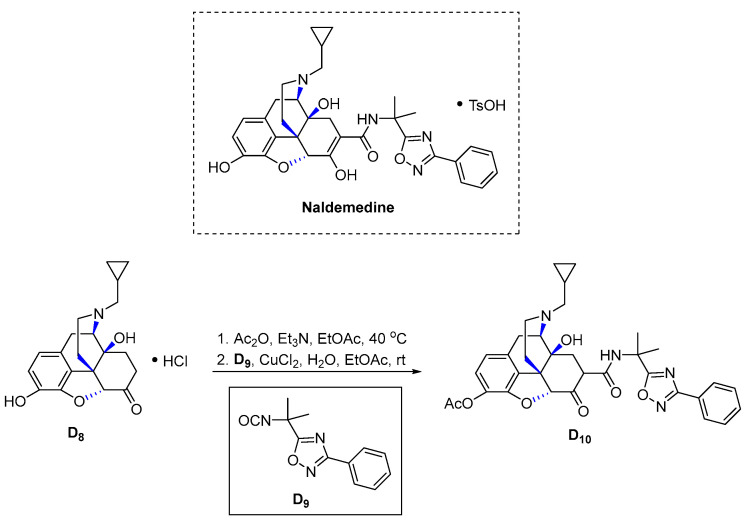
Synthesis of Naldemedine.

Shionogi & Co., Ltd. (Osaka, Japan) developed naldemedine for the treatment of opioid-induced constipation [144]. In addition to naturally occurring compounds initiating chiral synthesis, commercially available naltrexone hydrochloride (**D_8_**) affords naldemedine tosylates in 66% yield (Scheme 55) [145].

#### 3.4.4. Valbenazine (2017)

**Scheme 56:**
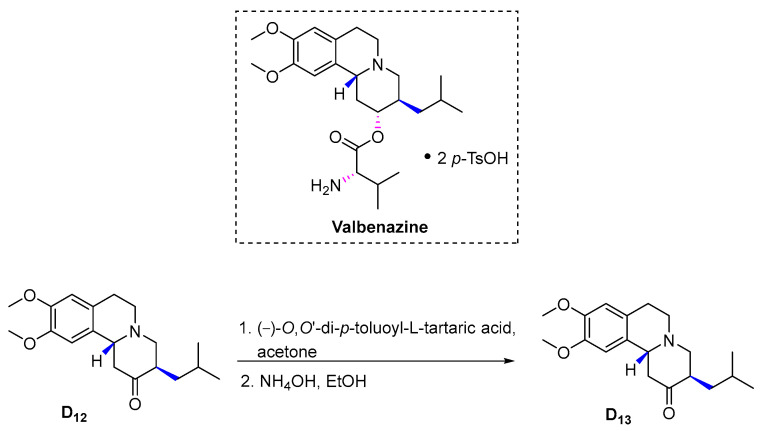
Synthesis of Valbenazine.

Neurocrine Biosciences developed valbenazine for the treatment of tardive dyskinesia in adults [146]. The well-known resolving agent L-tartaric acid (DPTTA) is used to separate (±)-tetrabenazine (**D_12_**) to yield (+)-amine **D_13_**, which is essential for the formation of valbenazine (Scheme 56) [147].

#### 3.4.5. Eravacycline (2018)

**Scheme 57:**
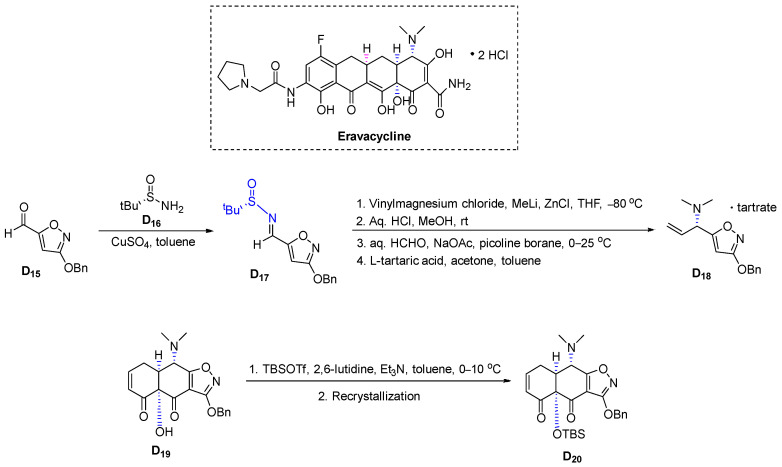
Synthesis of Eravacycline.

Eravacyclin, a tetracycline, is used to treat complicated intra-abdominal infections. Tetraphase Pharmaceuticals discovered and developed eravacycline [148,149,150]. Eravacycline is composed of a chiral isoxazole fragment, **D_20_**, and a dibenzyl amine derivative. The **D_20_** preparation involves the two following methods (Scheme 57) [151]:

1. Ellman sulfinamide auxiliary (**D_16_**) is used to convert aldehyde (**D_15_**) to sulfinimine (**D_17_**), which eventually leads to the formation of the chiral tartarate derivative, **D_18_**;
2. Recrystallization with isopropanol provides pure chiral tricyclic fragment D_20_ from the corresponding enone derivative, **D_19_**.

#### 3.4.6. Sarecycline Hydrochloride (2018)

**Scheme 58:**
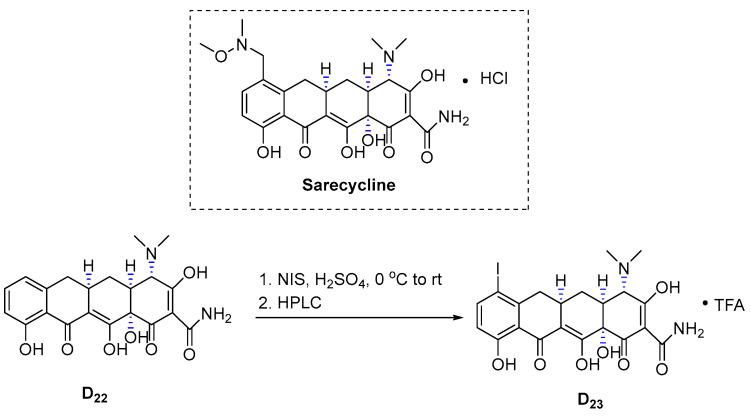
Synthesis of Sarecycline.

Paratek Pharmaceuticals discovered sarecycline; however, the drug was solely developed by Allergan. It belongs to the tetracycline class of antibiotics and is used for the treatment of inflammatory lesions of acne vulgaris [152]. A semi-synthetic tetracycline antibiotic, sancycline (**D_22_**), upon iodination with N-iodosuccinimide and further purification provided the iodosancycline salt, **D_23_**, is required for the synthesis of sarecycline hydrochloride (Scheme 58) [153,154,155].

#### 3.4.7. Omadacycline (2018)

**Scheme 59:**
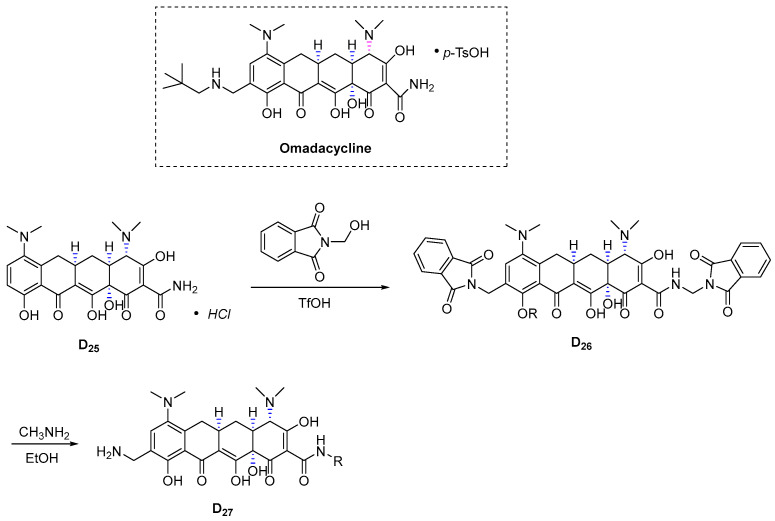
Synthesis of Omadacycline.

Discovered and developed by Paratek Pharmaceuticals [156], omadacycline, a tetracycline antibiotic, is used to treat acute bacterial skin infections and community-acquired pneumonia. Analogous to sarecycline, the tetracyclic antibiotic drug minocycline (**D_25_**) initiates the synthesis of omadacycline. Condensation with N-(hydroxymethyl)phthalimide in the presence of triflic acid affords a mixture of **D_26_** upon hydrolysis with methyl amine to generate the chiral intermediate, **D_27_** (Scheme 59) [157].

#### 3.4.8. Vibegron (2018)

**Scheme 60:**
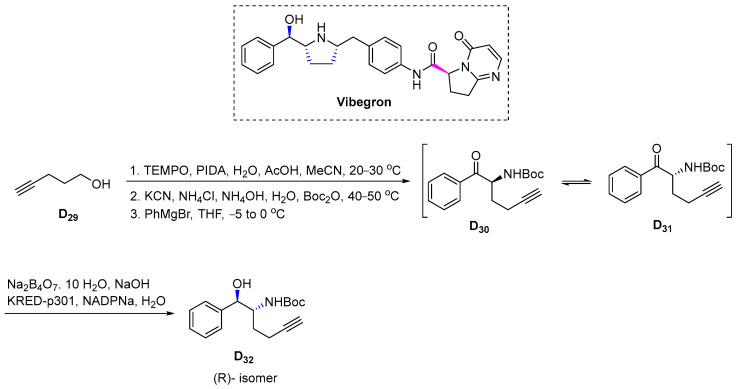
Synthesis of Vibegron.

Vibegron was discovered by Merck and developed by Kyorin Pharmaceutical Co., Ltd. (Tokyo, Japan) and Kissei Pharmaceutical Co., Ltd. (Matsumoto, Japan). It is used for the treatment of overactive bladder [158]. The key step in the synthesis of vibegron is to facilitate both the epimerization and reduction of racemic mixtures **D_30_** and **D_31_**. Ketoreductase and the cofactor NADPNa are used to form the (R)-isomer **D_32_** (Scheme 60) [159].

#### 3.4.9. Ubrogepant (2019)

**Scheme 61:**
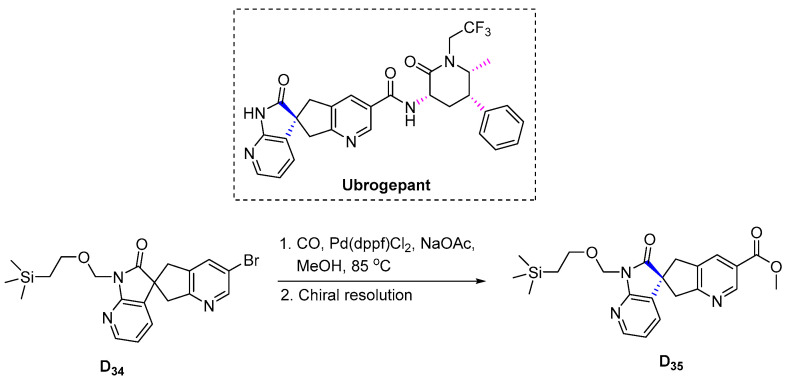
Synthesis of Ubrogepant.

Ubrogepant was developed by Allergan, Inc. It is used to treat migraines in adults [160]. Resolution by means of SFC separation, focused on racemic spiro intermediate **D_34_** resulted in the formation of mono-configurational fragment **D_35_**, as depicted in Scheme 61 [161].

#### 3.4.10. Cedazuridine (2020)

**Scheme 62:**
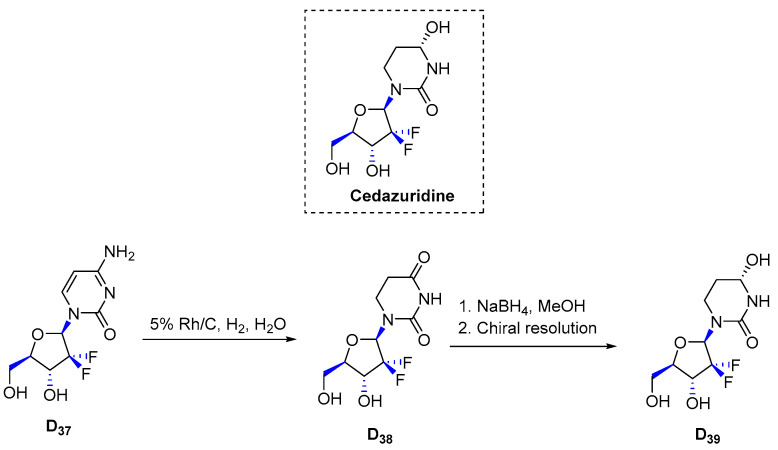
Synthesis of Cedazuridine.

Cedazuridine, in combination with decitabine, is used for the treatment of myelodysplastic syndrome [162]. Chiral chromatographic separation of racemic intermediate **D_38_** affords the chiral cedazuridine, **D_39_**, in a three-step synthesis (Scheme 62) [163].

#### 3.4.11. Vibegron (2020)

**Scheme 63:**
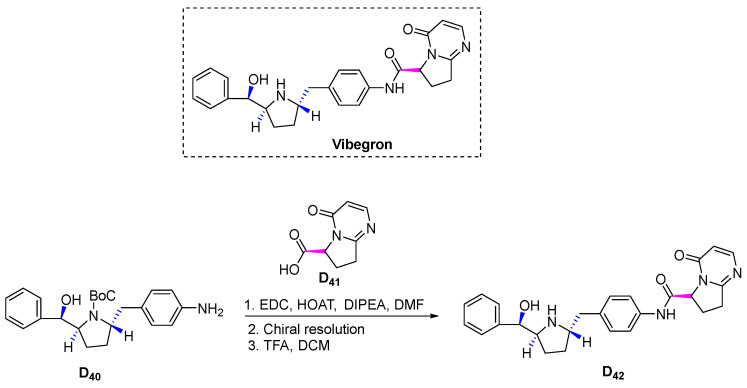
Synthesis of Vibegron.

Vibegron is used to treat overactive bladder with symptoms, such as urge urinary incontinence and urinary frequency [158]. Large-scale synthesis of the drugs is outlined in Scheme 63. Chiral amine, **D_40_**, generated from hexynoic acid, undergoes acid-amine coupling with another chiral acid, **D_41_**, followed by chiral SFC separation, to give the drug in pure form (Scheme 63) [164].

### 3.5. Drugs with Five Chiral Centers

#### 3.5.1. Narlaprevir (2016)

**Scheme 64:**
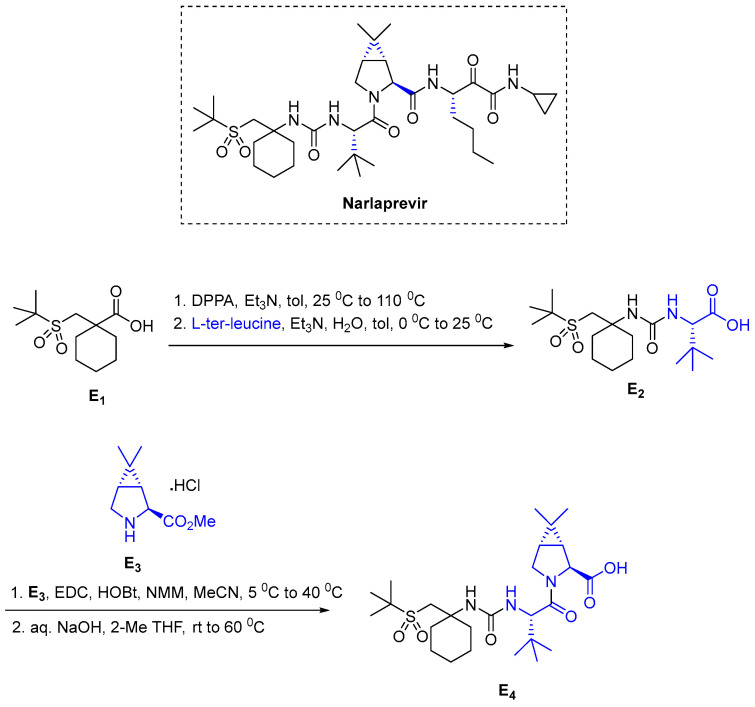
Synthesis of Narlaprevir.

Narlaprevir was developed by Schering-Plough Corporation and the Texas Liver Institute and succeeded by R-Pharm Pharmaceuticals. It is an anti-infective drug used for the treatment of hepatitis C virus (HCV) genotype 1 infections [165]. The kilogram-scale synthesis of narlaprevir is presented in Scheme 64 [166,167]. Chirality is induced during the conversion of the acid intermediate **E_1_** to the urea derivative, **E_2_**, in the presence of leucine. Another three chiral centers from the bicyclic amine, **E_3_**, are introduced by peptide coupling conditions.

#### 3.5.2. Elbasvir (2016)

**Scheme 65:**
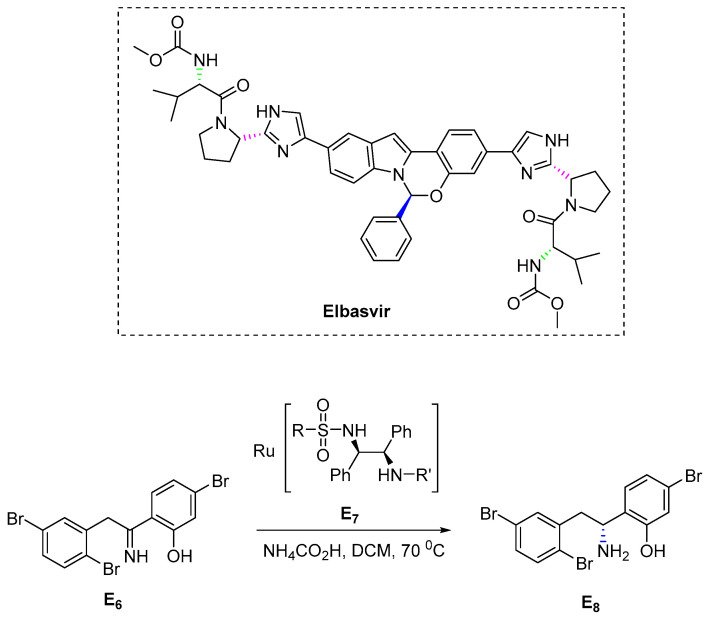
Synthesis of Elbasvir.

Merck developed the combination of elbasvir and grazoprevir for the treatment of chronic HCV infections [168]. Elbasvir, which contains five chiral carbons, is shown in Scheme 65. The key step in the synthesis of elbasvir is the asymmetric reduction of imine derivative **E_6_** in the presence of a ruthenium catalyst, **E_7_** [169].

#### 3.5.3. Latanoprostene Bunod (2017)

**Scheme 66:**
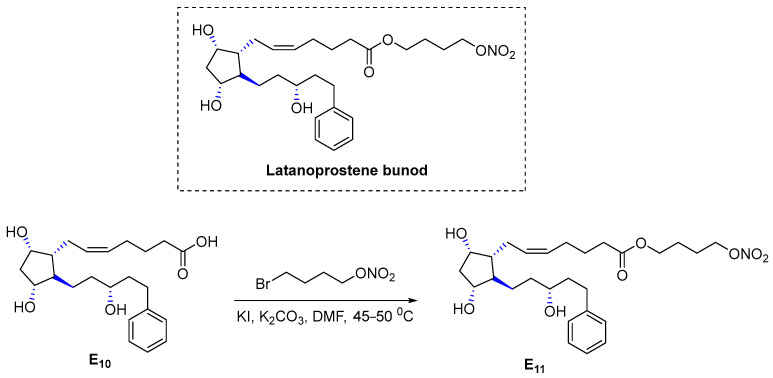
Synthesis of Latanoprostene bunod.

Latanoprostene bunod was discovered by the NicOx–Pfizer collaboration and developed by Bausch and Lomb. It is used to reduce intraocular pressure in patients with open-angle glaucoma or ocular hypertension [170]. In this chiral pool approach, latanoprost acid (**E_10_**) is treated with 4-bromobutyl nitrate in the presence of K_2_CO_3_ and KI (Scheme 66) [171,172].

#### 3.5.4. Danoprevir (2018)

**Scheme 67:**
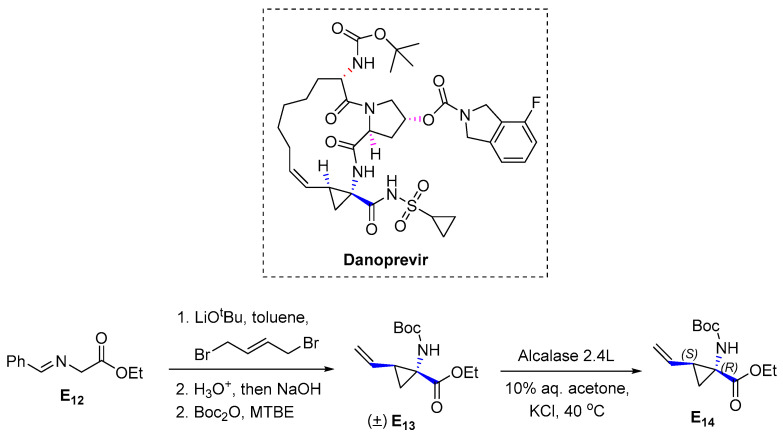
Synthesis of Danoprevir.

InterMune Inc. and Array Biopharma Inc. developed danoprevir for the treatment of non-cirrhotic genotype 1b chronic HCV infections [173]. Dialkylation of imine intermediate **E_12_** with 2-butene-1,4-dibromide afforded racemic vinylcyclopropane **E_13_**, which upon enzymatic resolution with alcalase 2.4L resulted in the formation of desired enantiomer **E_14_**. **E_14_** is the key chiral fragment in the synthesis of danoprevir (Scheme 67) [174].

### 3.6. Drugs with More Than Five Chiral Centers

For drugs with more than five chiral centers, only the step in which chirality is induced is described in the scheme.

#### 3.6.1. Velpatasvir (2016)

**Scheme 68:**
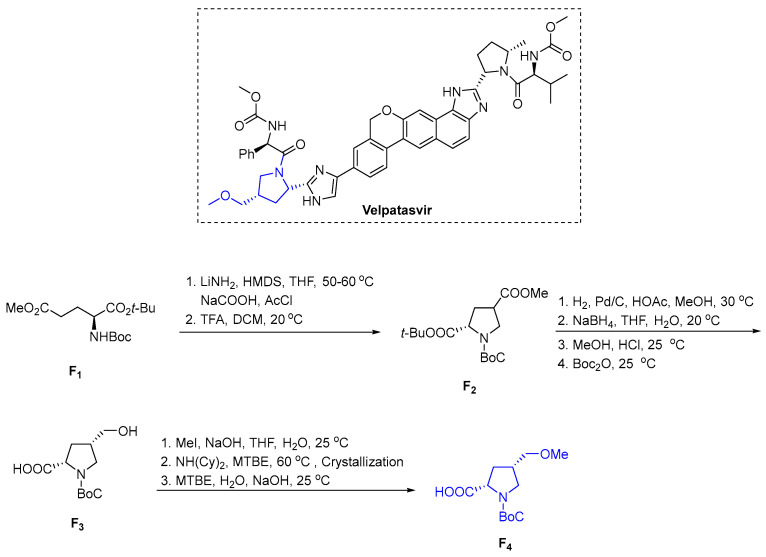
Synthesis of Velpatasvir.

Velpatasvir, an antiviral drug developed by Gilead Sciences, is used, in combination with sofosbuvir, for the oral treatment of chronic HCV genotypes (1–6) [175]. The combination of chiral subunits in velpatasvir is shown in Scheme 68 [176]. Of all the chiral centers present in the drug, only the initial chiral induction by means of crystallization is highlighted. Commercially available glutamate (**F_1_**) undergoes intramolecular condensation to form dihydropyrrole (**F_2_**). The reduction of ester functionalities, followed by alkylation and crystallization, results in the cis form, **F_4_**.

#### 3.6.2. Obeticholic Acid (2016)

**Scheme 69:**
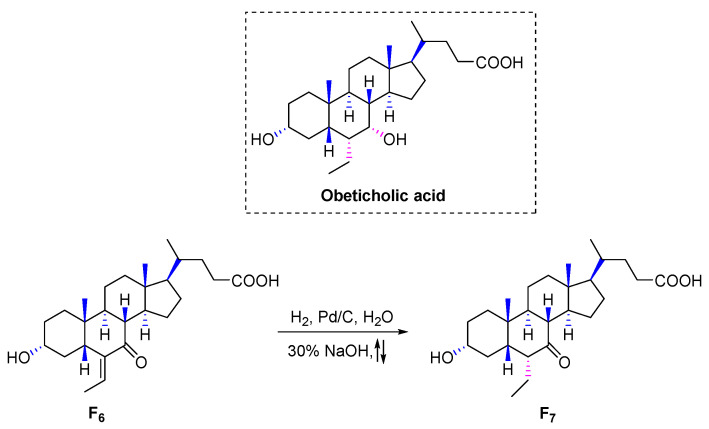
Synthesis of Obeticholic acid.

Obeticholic acid, in combination with ursodeoxycholic acid, is used in the treatment of primary biliary cholangitis [177]. This drug was discovered at the Universita de Perugia and developed by Intercept Pharmaceuticals. In this chiral pool strategy, hydrogenation of the olefin intermediate **F_6_**, followed by heating to reflux, resulted in the epimerized fragment α-ethyl ketone **F_7_** (Scheme 69) [178,179].

#### 3.6.3. Grazoprevir Hydrate (2016)

**Scheme 70:**
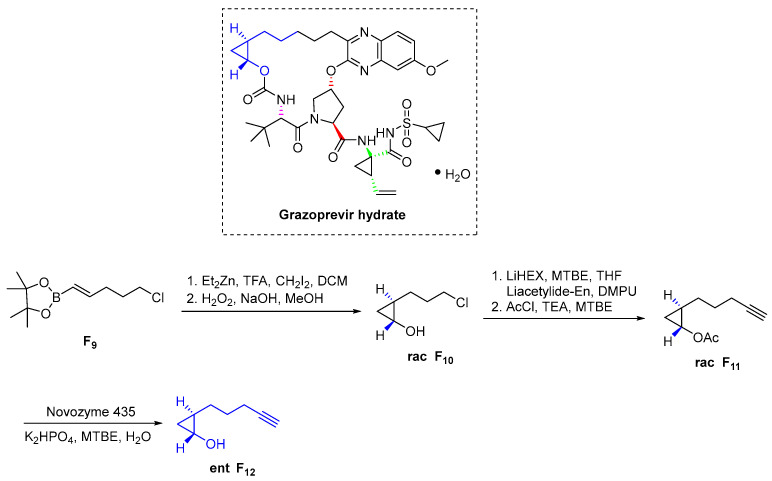
Synthesis of Grazoprevir hydrate.

Merck discovered grazoprevir hydrate, a drug that, in combination with elbasvir, has been used to treat HCV infections. Similar to danoprevir, the key chiral subunit, **F_12_**, is formed by enzymatic resolution of the racemic cyclopropyl intermediate, **F_11_**. Commercially available boronate (**F_9_**) is converted to the cyclopropane derivative, **F_10_**, under Simmons–Smith conditions. Finally, the enantiomer is produced by resolution with a novozyme, followed by crystallization [180]. The remaining subunits are successively inserted in a step-by-step sequence (Scheme 70).

#### 3.6.4. Ertugliflozin-l-pyroglutamic Acid (2017)

**Scheme 71:**
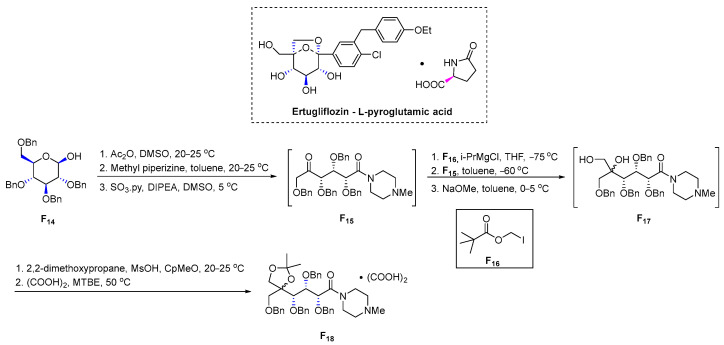
Synthesis of Ertugliflozin-l-pyroglutamic acid.

Ertugliflozin, discovered by Pfizer and co-developed by Pfizer and Merck, is used for the treatment of type II diabetes mellitus [181]. The synthesis of ertuglifozin commences from a chiral pool of glucose derivative **F_14_** (Scheme 71) [182]. Oxidation, amidation, and treatment under Parikh–Doering conditions (SO_3_. Py) affords the chiral intermediate, **F_15_**. Oxalate salt (**F_18_**) formation occurs via a series of reactions, viz., pivalate group removal and protection of the hindered OH group.

#### 3.6.5. Voxilaprevir (2017)

**Scheme 72:**
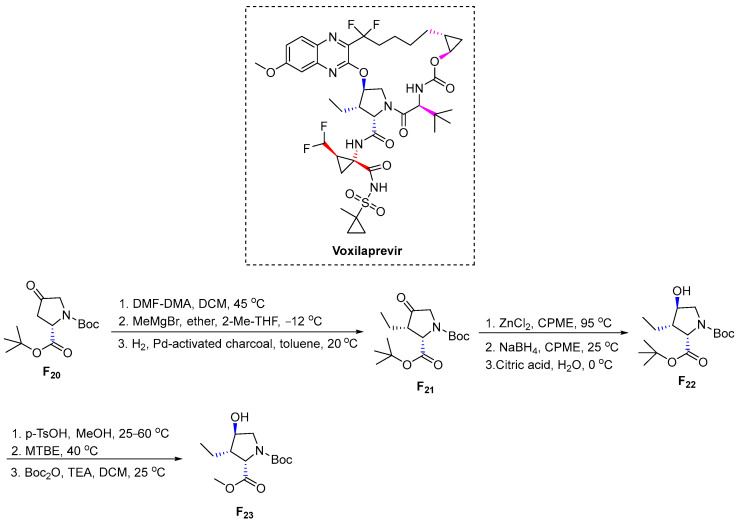
Synthesis of Voxilaprevir.

Voxilaprevir, in combination with sofosbuvir and velpatasvir, is used to treat chronic HCV genotypes [183]. Gilead described the synthesis of voxilaprevir in nine steps from the pyrrolidinol derivative, **F_23_**. The enone formed by the reaction of pyrrolidinone (**F_20_**) with Grignard reagent, followed by hydrogenation, results in the addition of an ethyl group on the pyrrolidine ring (**F_21_**). Reduction, subjection to citric acid, and Boc protection introduce three stereocenters to the alcohol derivative, **F_23_**. Chiral centers are inserted in such a way that three are from the pyrrolidinol fragment (**F_20_**); three are from the cyclopropyl fragment, and two are from the cyclopropyl amine fragment (Scheme 72) [184].

#### 3.6.6. Pibrentasvir (2017)

**Scheme 73:**
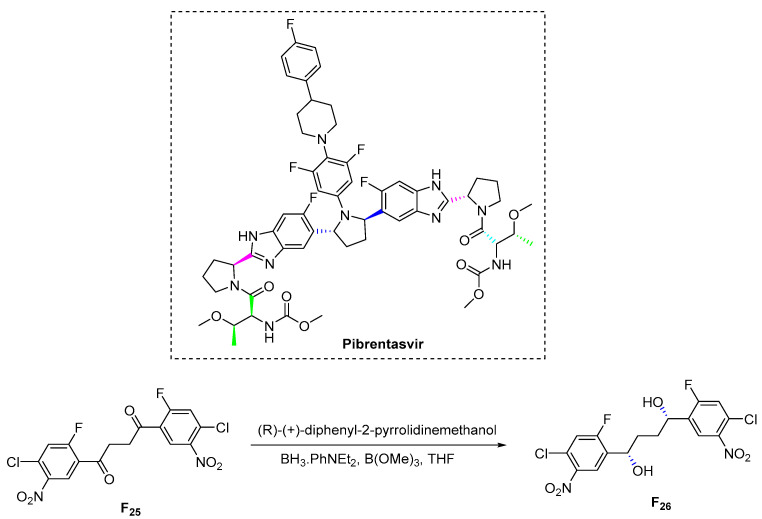
Synthesis of Pibrentasvir.

Pibrentasvir, in combination with glecaprevir, was discovered and developed by Abbvie and Enanta Pharmaceuticals and is used for the treatment of chronic HCV genotypes (1–6) [185]. Pibrentasvir is formed via the union of different chiral subunits at various stages of synthesis. The very first stereogenic formation is initiated by the chiral diol fragment, **F_26_**, which was synthesized by asymmetric bis reduction in the presence of (R)-(+)-α,α-diphenyl-2-pyrrolidinemethanol by the Corey–Bakshi–Shibata (CBS) reduction mechanism (Scheme 73) [186].

#### 3.6.7. Glecaprevir (2017)

**Scheme 74:**
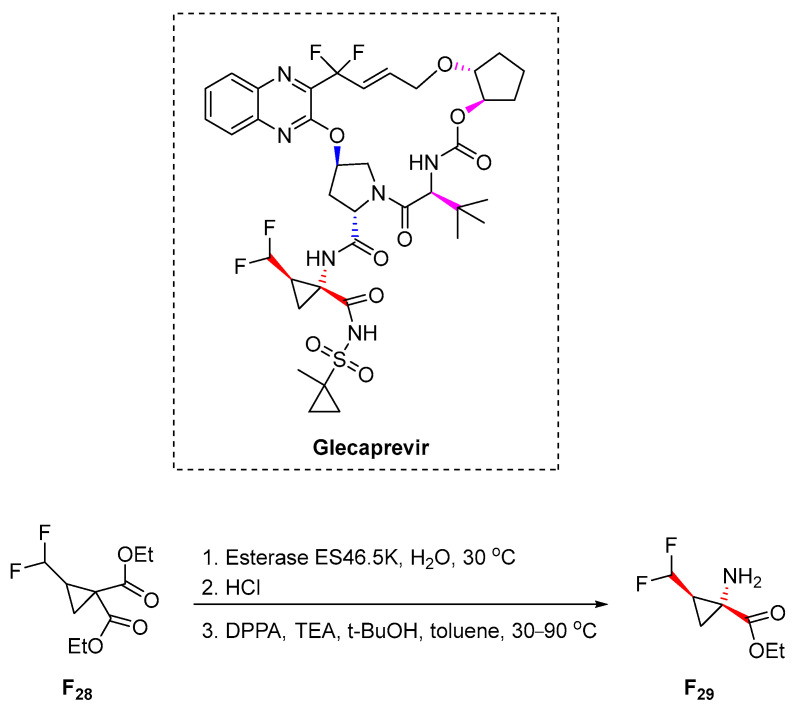
Synthesis of Glecaprevir.

Glecaprevir was first discovered and developed by Abbvie and Enanta Pharmaceuticals. Glecaprevir, in combination with pibrentasvir, has been used to treat chronic HCV infections. Glecaprevir is an assembly of chiral fragments. Of these, one notable step is the resolution of the cyclopropane intermediate, **F_28_**, utilizing esterase as a resolving agent (Scheme 74) [187].

#### 3.6.8. Segesterone Acetate (2018)

**Scheme 75:**
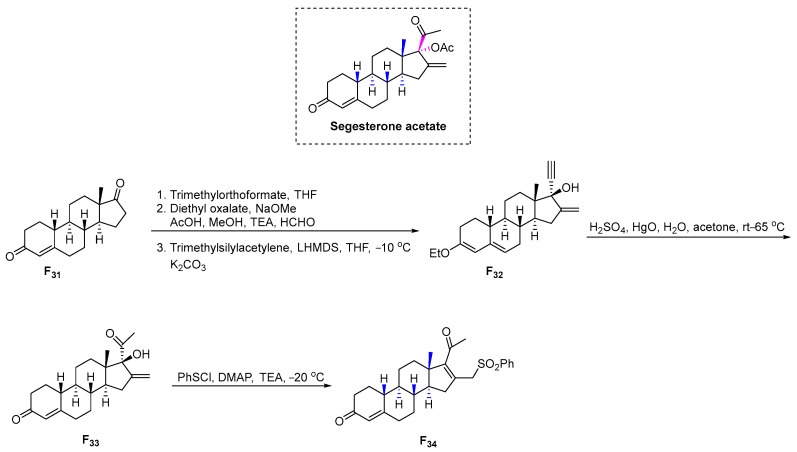
Synthesis of Segesterone acetate.

The Population Council developed segesterone acetate, a progestin hormonal contraceptive. The chiral pool substrate, 19-norandrostenedione (**F_31_**), is converted into an alcohol intermediate (**F_33_**) through the hydration of the alkyne derivative, **F_32_**. Inversion of the configuration is observed at C-17 (**F_34_**) via 2,3-sigmatropic rearrangement (Scheme 75) [188].

#### 3.6.9. Plitidepsin (2018)

**Scheme 76:**
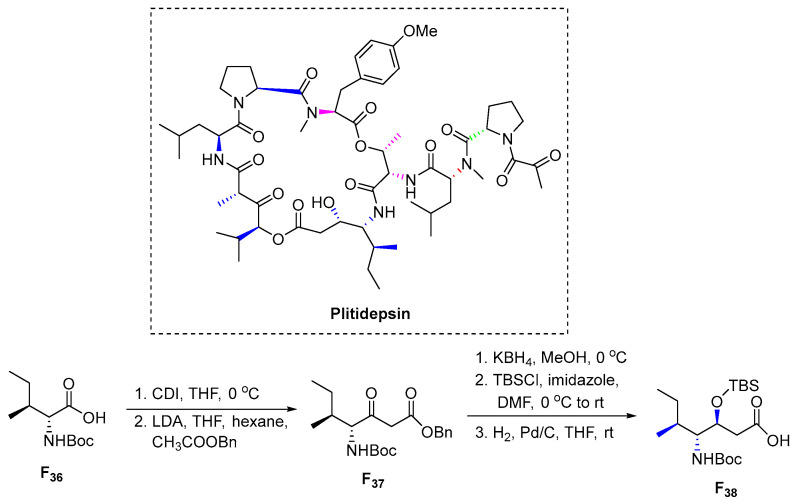
Synthesis of Plitidepsin.

Plitidepsin, a natural marine product, is a potent antiproliferative drug [189]. The combination of different subunits comprises the synthesis of plitidepsin in seven steps. The chiral fragment necessary for the synthesis of the drug is generated starting from the conversion of N-Boc isoleucine **F_36_** to keto ester **F_37_**, followed by the sequential reduction, silylation, and deprotection of the ester to give plitidepsin carboxylic acid **F_38_** in good yield (Scheme 76) [190].

#### 3.6.10. Moxidectin (2018)

**Scheme 77:**
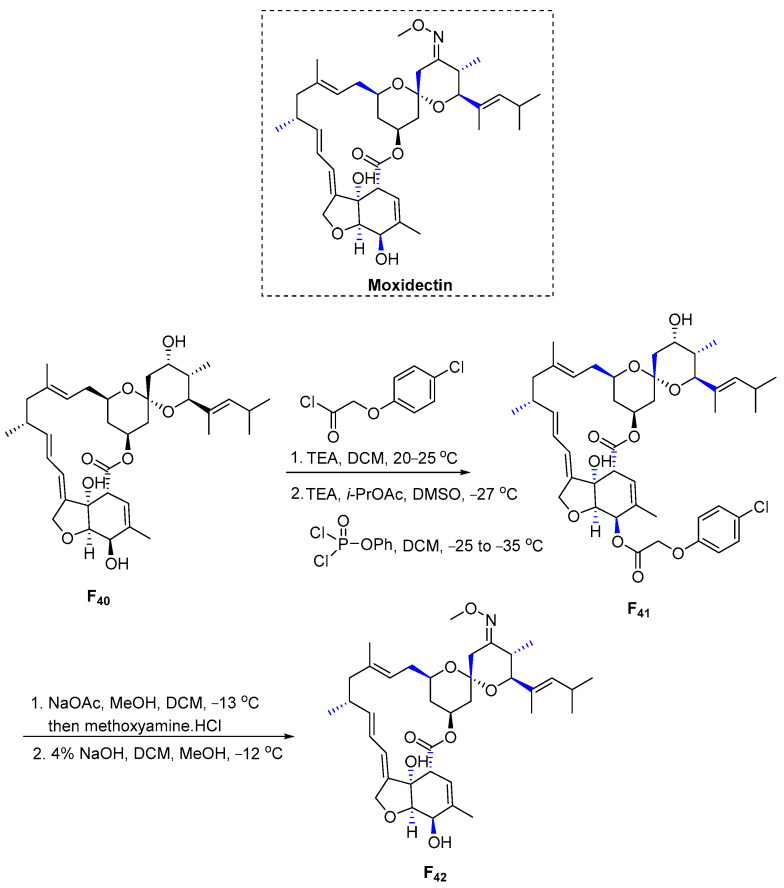
Synthesis of Moxidectin.

The Medicines Development for Global Health (MDGH) developed moxidectin. It is used for the treatment of onchocerciasis, also known as river blindness [191]. Moxidectin belongs to the milbemycin class comprised of 16-membered macrocyclic lactones. The chiral pool substrate nemadectin (**F_40_**), obtained by fermentation of *Streptomyces cyanogriseus* ssp. *noncyanogenus*, is converted to the ketone intermediate, **F_41_**, which then undergoes oxime formation and selective saponification of the ester-protecting group to afford moxidectin **F_42_**. It is worth mentioning that the oxime **F_42_** retains (*E*)-configuration throughout the last two steps. (Scheme 77) [192].

#### 3.6.11. Plazomicin (2018)

**Scheme 78:**
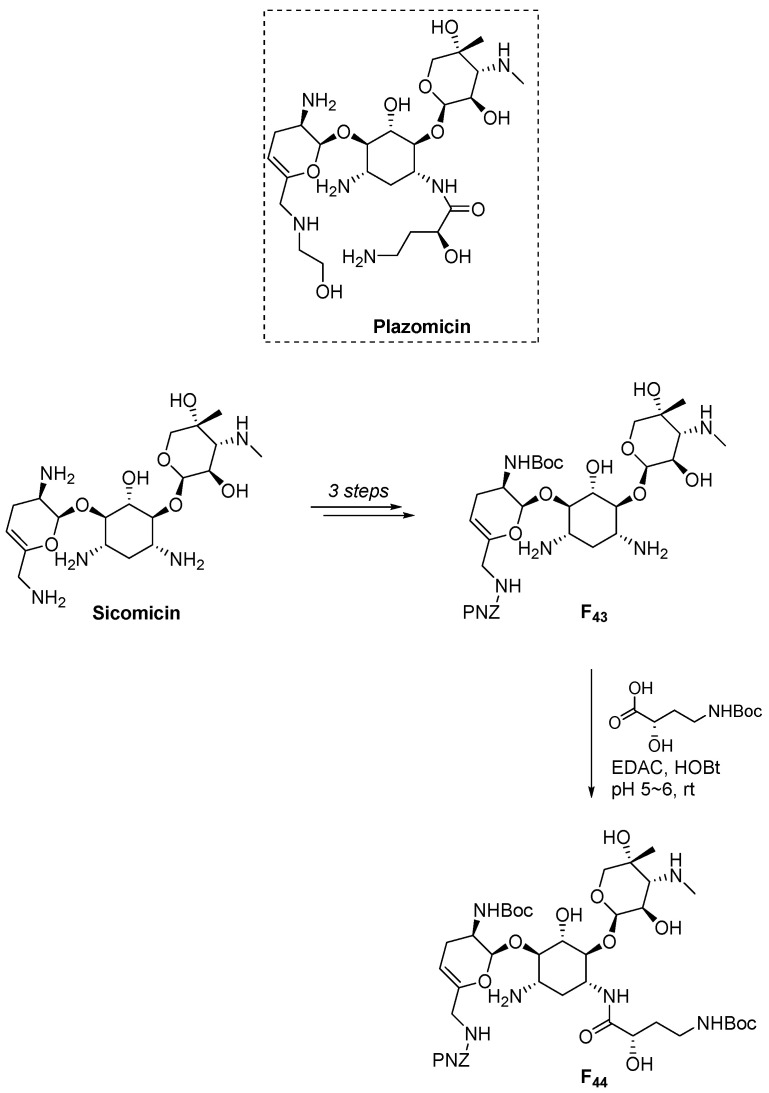
Synthesis of Intermediate of Plazomicin.

Ionis Pharmaceuticals, Inc. discovered plazomicin, which was then further developed by Achaogen. It is used for the treatment of complicated urinary tract infections [193]. Unlike the chiral pool syntheses, commercially available and natural sisomicin commences the synthesis of plazomicin. The key intermediate **F_44_** was synthesized by converting sisomicin to protected amine **F_43_** in three step sequences and incorporation of Boc-(S)-HABA to amine (Scheme 78) [194,195,196,197,198,199].

#### 3.6.12. Tecovirimat (2018)

**Scheme 79:**
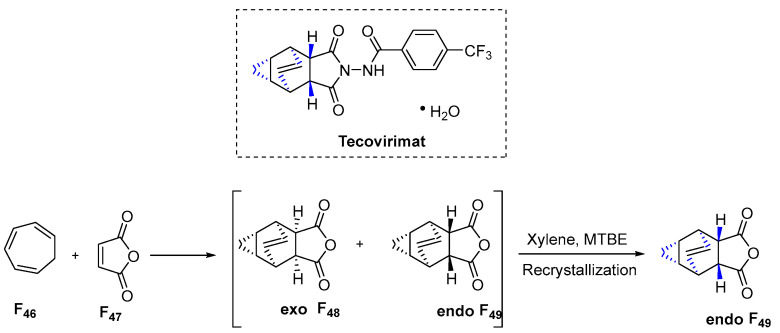
Synthesis of Tecovirimat.

SIGA Technologies and the United States Department of Health Services Biomedical Advances Research and Development Authority developed tecovirimat [200]. Cycloaddition of cycloheptatriene (**F_46_**) and maleic anhydride (**F_47_**) delivers mixtures **F_48_** and **F_49_**, which then undergo recrystallization with methyl *tert*-butyl ether (MTBE) to isolate the endo isomer, **F_49_** (Scheme 79) [201].

#### 3.6.13. Lefamulin (2019)

**Scheme 80:**
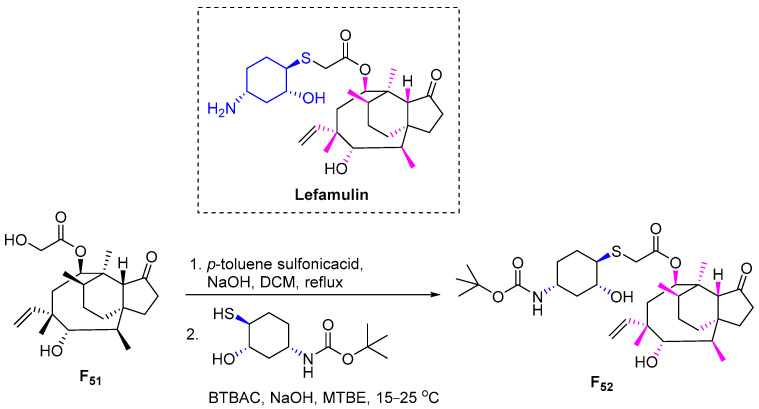
Synthesis of Lefamulin.

Nabriva Therapeutics developed lefamulin for the treatment of community-acquired bacterial pneumonia [202]. The total synthesis of lefamulin is performed in five steps, beginning with pleuromulin **F_51_**. The key subunit involved in the synthesis, **F_52_**, is obtained by the reaction with cyclohexanethiol in the presence of sodium hydroxide and benzyl(tributyl)ammonium chloride (BTBAC) in methyl tert-butyl ether (MTBE) (Scheme 80) [203].

#### 3.6.14. Afamelanotide (2019)

**Scheme 81:**
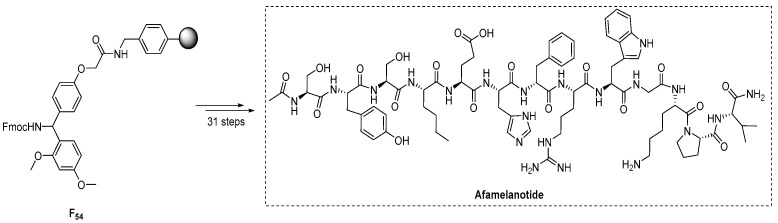
Synthesis of Afamelanotide.

Afamelanotide was developed by Clinuvel Pharmaceuticals. It is used to treat erythropoietic protoporphyria [204]. Afamelanotide, with the amino acid sequence Ac-Ser-Tyl-Ser-Nle-Glu-His-D-Phe-Arg-Trp-Gly-Lys-Pro-Val-NH_2_, is synthesized in 31 steps from the commercially available resin, **F_54_** (Scheme 81) [205,206].

#### 3.6.15. Brexanolone (2019)

**Scheme 82:**
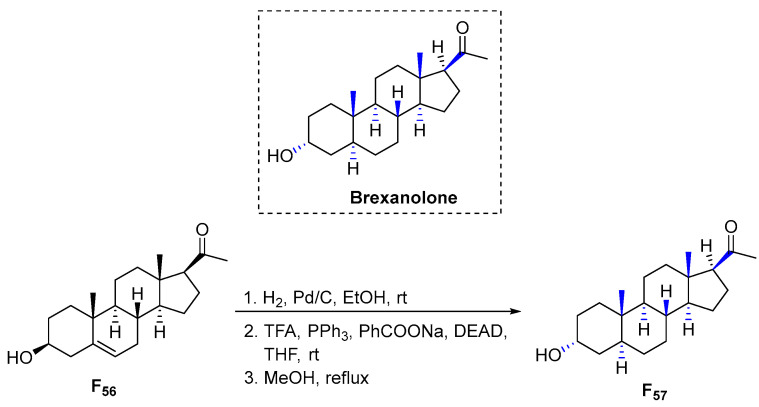
Synthesis of Brexanolone.

Sage Therapeutics developed brexanolone, which is used in the treatment of postpartum depression [207]. Pregnenolone (**F_56_**) initiates the linear synthesis of brexanolone (**F_57_**) in a sequential hydrogenation reduction and Mitsunobu reaction with diethylazodicarboxylate (DEAD) and triphenylphosphine (Scheme 82) [208].

#### 3.6.16. Bremelanotide (2019)

**Scheme 83:**
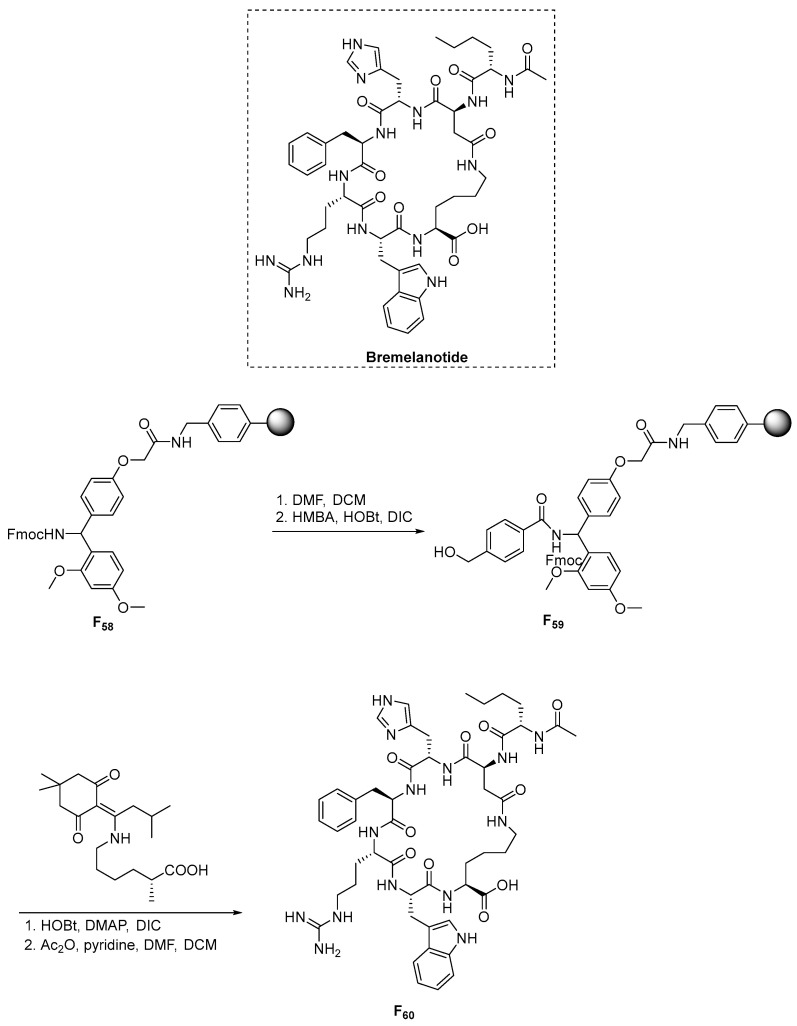
Synthesis of Bremelanotide.

Bremelanotide is used in the treatment of hypoactive sexual desire disorder in premenopausal women [209]. Similar to afamelanotide, the synthesis begins with Rink-Amide-AM-resin (**F_58_**) and continues with the successful linkage of chiral amino acid derivatives, removal of N-Fmoc protecting group, and condensation with Fmoc-Trp(Boc)-OH (Scheme 83) [210].

#### 3.6.17. Lurbinectedin (2020)

**Scheme 84:**
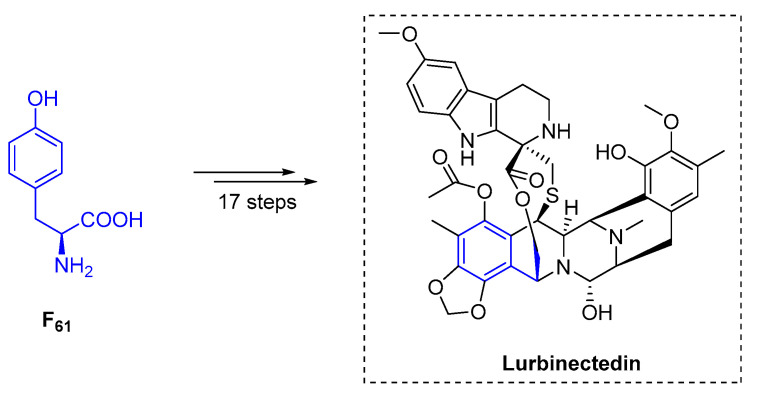
Synthesis of Lurbinectedin.

Lurbinectedin is used in the treatment of metastatic small-cell lung cancer in patients who have undergone platinum-based chemotherapy [211]. The total synthesis of lurbinectedin is carried out in 27 steps starting from the chiral pool l-tyrosine, **F_61_** (Scheme 84) [212].

#### 3.6.18. Lactitol (2020)

**Scheme 85:**
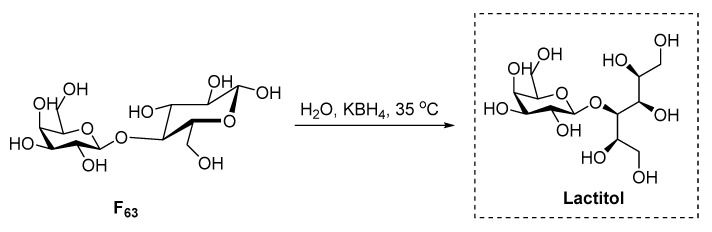
Synthesis of Lactitol.

Pizensy developed lactitol, a *β*-d-galactopyranosyl-d-glucitol. It is used to treat chronic idiopathic constipation [213]. The chiral pool synthesis of lactitol from lactose (**F_63_**) is achieved in a single step (Scheme 85) [214].

#### 3.6.19. Setmelanotide (2020)

**Scheme 86:**
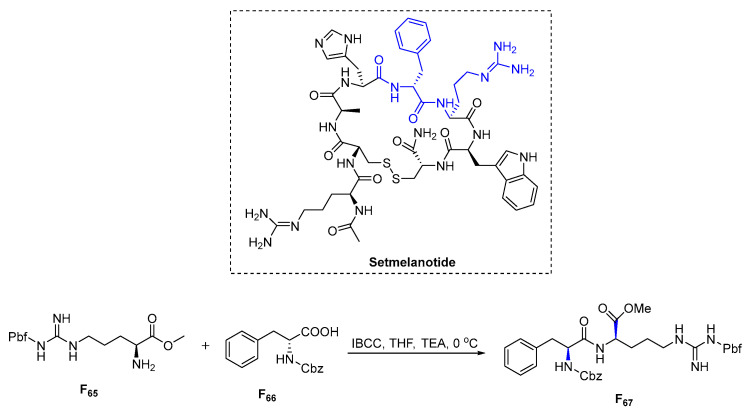
Synthesis of Setmelanotide.

Chronic weight management can be treated with setmelanotide [215]. Setmelanotide is synthesized in ten steps with successive insertion of chiral centers, starting from commercially available arginine (**F_65_**) and phenyl alanine (**F_66_**) derivatives (Scheme 86) [216].

#### 3.6.20. Clascoterone (2020)

**Scheme 87:**
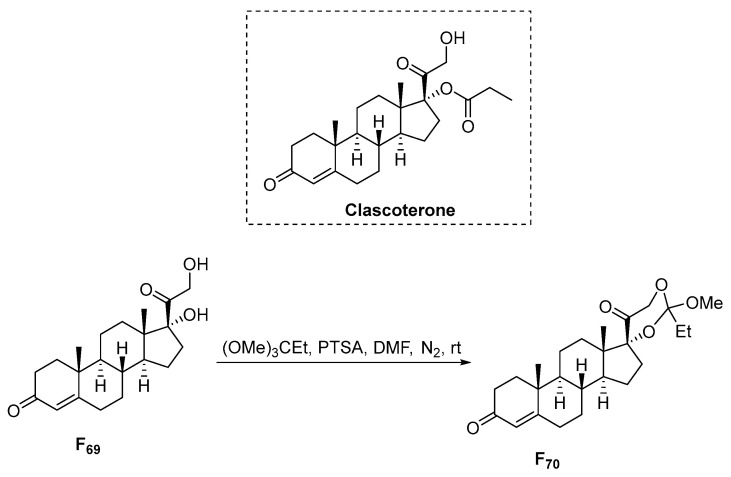
Synthesis of Clascoterone.

Cassiopea developed clascoterone. It is widely used for the treatment of acne [217]. Hydrocortisone (**F_69_**) induces chirality in clascoterone in two steps (Scheme 87) [218].

#### 3.6.21. Artesunate (2020)

**Scheme 88:**
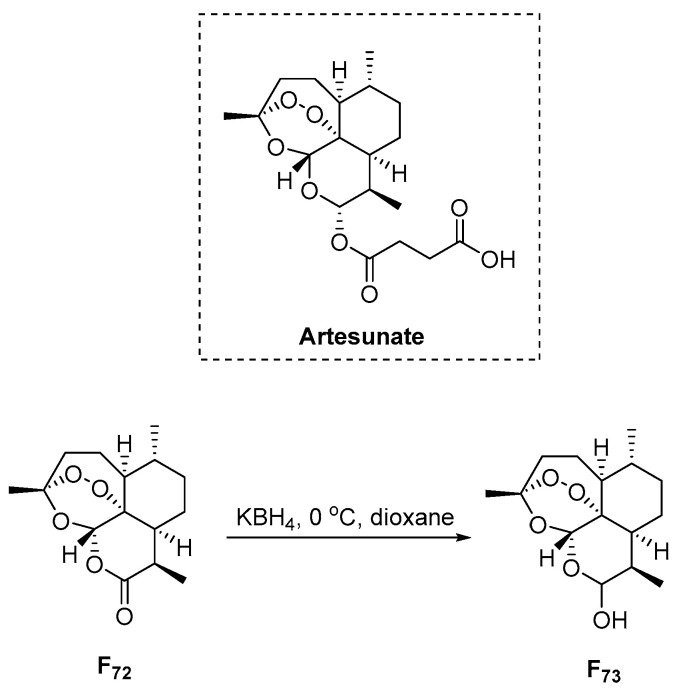
Synthesis of Artesunate.

Arnivas developed artesunate for the treatment of severe malaria. The semi-synthesis of artesunate is accomplished using a chiral pool of artesimin (**F_72_**) isolated from *Artemisia annua* (Scheme 88) [219].

#### 3.6.22. Remdesivir (2020)

**Scheme 89:**
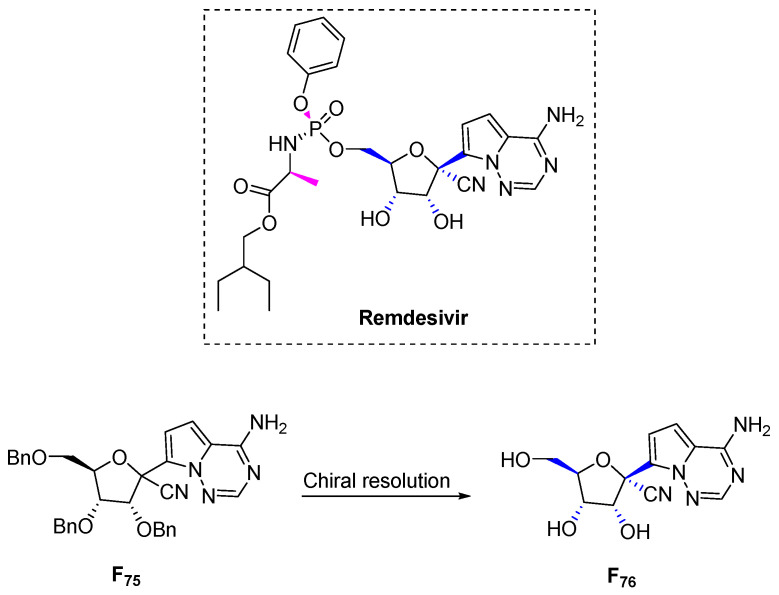
Synthesis of Remdesivir.

Remdesivir was developed by Gilead Sciences [220]. It is used for the treatment of Ebola virus disease. The triazine intermediate, **F_75_**, generated from the hemiacetal derivative undergoes chiral SFC separation to form chiral fragment **F_76_**, which is essential for the development of remdesivir drugs (Scheme 89) [221].

## 4. Conclusions

The concept of chirality has gained considerable attention owing to its importance in the field of medicinal chemistry. The importance of chirality is evidenced by the fact that most of the new drugs introduced annually into the market are single enantiomers. In general, drugs have structural homology across similar biological targets, and it is widely believed that knowledge of new chemical entities and approaches to their construction will enhance our ability to discover new drugs more efficiently. Incorporating chirality into drug discovery is a promising approach to better engage biological targets with enhanced drug properties. Chirality has the efficacy to remedy the challenges of drug optimization by exploiting the three-dimensional nature of biology. Moreover, chiral small molecules are upsurging as an attractive clinical advantage in drug discovery. Our quest to find the way by which chirality is induced led us to summarize the asymmetric synthesis of 89 FDA drugs approved from 2016 to 2020. The majority of the drugs were employed for the treatment of infectious diseases (28 drugs), oncology (20 drugs), metabolic and gastrointestinal disorders (14 drugs), central nervous system disorders (12 drugs), and others (15 drugs). With 89 new drugs approved by the FDA in hand, we extracted a subgroup of small molecules featuring one or more chiral centers, and analyzed their synthetic profile. The recent major progress in new asymmetric synthetic methodologies and enantiomeric separation techniques encourage the effort of chiral drug development. With regard to the methods routinely utilized to check the enantiomeric purity at the different stages of the discovery process, the term “chiral SFC” proved to emerge in the list of the synthesized drugs. Furthermore, we found out that all the drugs have been approved as single enantiomers with a well-defined absolute stereochemistry.

It has since become clear that the individual enantiomers can have vastly different effects on the body, leading to a shift toward the development of single enantiomers as drugs. This trend is driven by advances in analytical techniques that allow for the separation and characterization of individual enantiomers, as well as a growing understanding of the pharmacology of chiral compounds. Additionally, the increasing focus on personalized medicine and the development of targeted therapies has led to a greater appreciation of the importance of chirality in drug discovery and development. Chiral drugs are now a major area of research and development in the pharmaceutical industry, with many companies investing heavily in the discovery and development of new chiral drugs. In terms of the costs of drug substances, the asymmetric synthesis of chiral drugs is still expensive. So, technological advances in asymmetric synthesis and chiral resolution/separation are continuously required. 

## Data Availability

Not applicable.
